# Rice H2A.Z negatively regulates genes responsive to nutrient starvation but promotes expression of key housekeeping genes

**DOI:** 10.1093/jxb/ery244

**Published:** 2018-06-28

**Authors:** Sara Zahraeifard, Maryam Foroozani, Aliasghar Sepehri, Dong-Ha Oh, Guannan Wang, Venkata Mangu, Bin Chen, Niranjan Baisakh, Maheshi Dassanayake, Aaron P Smith

**Affiliations:** 1 Department of Biological Sciences, Louisiana State University, Baton Rouge, LA, USA; 2 Department of Chemistry, Louisiana State University, Baton Rouge, LA, USA; 3 School of Plant, Environmental and Soil Sciences, Louisiana State University Agricultural Center, Baton Rouge, LA, USA

**Keywords:** ARP6-RNAi Knock-down, chromatin, genomics, H2A.Z, Pi deficiency, rice, *Oryza sativa*

## Abstract

The H2A.Z histone variant plays a role in the modulation of environmental responses, but the nature of the associated mechanisms remains enigmatic. We investigated global H2A.Z deposition and transcriptomic changes in rice (*Oryza sativa*) upon exposure to phosphate (Pi) deficiency and in response to RNAi knockdown of *OsARP6*, which encodes a key component of the H2A.Z exchange complex. Both Pi deficiency and *OsARP6*-knockdown resulted in similar, profound effects on global H2A.Z distribution. H2A.Z in the gene body of stress-responsive genes was negatively correlated with gene expression, and this was more apparent in response to Pi deficiency. In contrast, the role of H2A.Z at the transcription start site (TSS) was more context dependent, acting as a repressor of some stress-responsive genes, but an activator of some genes with housekeeping functions. This was especially evident upon *OsARP6*-knockdown, which resulted in down-regulation of a number of genes linked to chloroplast function that contained decreases in H2A.Z at the TSS. Consistently, *OsARP6*-RNAi plants exhibited lower chlorophyll content relative to the wild-type. Our results demonstrate that gene body-localized H2A.Z plays a prominent role in repressing stress-responsive genes under non-inductive conditions, whereas H2A.Z at the TSS functions as a positive or negative regulator of transcription.

## Teaser

In rice, gene body-localized H2A.Z plays a prominent role in repressing stress-responsive genes under non-inductive conditions, whereas H2A.Z at the transcription start site functions as a positive or negative regulator of transcription.

## Introduction

The organization of eukaryotic DNA into chromatin is a key determinant of DNA-related processes (Workman and Buchman, 1993). Thus, various interconnected mechanisms regulate DNA accessibility, including alteration of nucleosome structure via histone post-translational modifications (Zhang and Pugh, 2011) and replacement of canonical histones with specialized histone variants (Bönisch and Hake, 2012). Histone variants are encoded by non-allelic paralogous genes of core histones, but their deposition is replication-independent, making them adaptable to respond to external signals (Talbert and Henikoff, 2014). Indeed, the conserved H2A.Z histone variant is a key modulator of environmental responses. In yeast (*Saccharomyces cerevisiae*), H2A.Z is necessary for proper expression of nutrient-related genes (Adam *et al.*, 2001; Wan *et al.*, 2009), supporting a positive role for H2A.Z by poising genes for activation. Work in *Drosophila* has shown that +1 nucleosomes are a major barrier to transcription, but are less so when they contain H2A.Z. This points to a general role for H2A.Z in regulating gene expression via modulation of DNA accessibility (Weber *et al.*, 2014). Consistently, H2A.Z has been shown to facilitate assembly of both activating and repressive complexes at enhancers and promoters in murine embryonic stem cells (Hu *et al.*, 2013).

Studies in Arabidopsis have implicated H2A.Z in the regulation of diverse processes and responses in plants. Mutation of Arabidopsis *ACTIN RELATED PROTEIN 6* (*ARP6*), which encodes a key component of the Swi/Snf2-related (SWR1) complex that exchanges H2A.Z/H2B dimers for H2A/H2B dimers (March-Díaz and Reyes, 2009), reduces H2A.Z abundance at the *FLOWERING LOCUS C* (*FLC*) floral repressor locus, resulting in down-regulation of FLC and an early flowering phenotype (Deal *et al.*, 2007). This indicates a positive role for H2A.Z in transcriptional regulation in plants. Subsequent genome-wide examination of H2A.Z in Arabidopsis showed preferential deposition of H2A.Z near the transcription start site (TSS) of genes, and Zilberman *et al.* (2008) suggested that H2A.Z facilitates transcriptional activity. In contrast to supporting a positive role for H2A.Z, several studies have implicated H2A.Z in the repression of responses to environmental stressors, including pathogen challenge (March‐Díaz *et al*., 2008; Berriri *et al.*, 2016), phosphate (Pi) deficiency (Smith *et al.*, 2010), heat (Kumar and Wigge, 2010; Cortijo *et al.*, 2017), and drought (Sura *et al.*, 2017). These findings were supported by a global study showing a correlation between H2A.Z enrichment across gene bodies and lower transcription levels (but higher gene responsiveness) in genes mainly associated with responses to environmental and developmental stimuli (Coleman-Derr and Zilberman, 2012). Recently, in another genome-wide examination of H2A.Z localization in Arabidopsis, a dual role for H2A.Z in transcriptional regulation was proposed by Sura *et al.* (2017). Their model suggested that H2A.Z has a repressive effect on transcription when localized in gene bodies, but is important in maintaining the transcriptional activity of some genes when localized to +1 nucleosomes. On the other hand, a separate report suggested a repressive role for H2A.Z at +1 nucleosomes by establishing low gene accessibility, and also suggested repression of enhancer activity by H2A.Z deposition (Dai *et al.*, 2017).

Compared to Arabidopsis, investigations of H2A.Z function in other plant species are lacking. A single recent report on H2A.Z localization in rice (*Oryza sativa*) indicated distinct H2A.Z genic profiles between callus and seedling samples, suggesting a role for H2A.Z in tissue development (Zhang *et al.*, 2017). The study also showed differential associations among H2A.Z and other chromatin marks at distinct genic regions, as has been shown for Arabidopsis (Zilberman *et al.*, 2008; Dai *et al.*, 2017). Despite these advances, investigations of H2A.Z in response to environmental stimuli and the impact of loss of H2A.Z on transcription have not been reported for rice, a global staple crop that feeds billions. In addition, more understanding on the role of H2A.Z in transcriptional regulation in plants is needed to reconcile the seemingly contradictory findings from previous studies. In this study we examined the role of H2A.Z in modulating gene expression in rice by investigating global H2A.Z deposition and associated transcriptomic changes upon exposure to deficiency of a major nutrient (i.e. Pi) and/or in response to RNA interference-mediated knockdown of *OsARP6*. Our results demonstrate that Pi deficiency and *OsARP6*-knockdown have similar, profound effects on H2A.Z localization across rice genes. Our findings also reveal that distinct subsets of genes exhibit differential sensitivities to altered expression upon H2A.Z perturbation, which correlate with gene function and genic location of H2A.Z.

## Materials and methods

### Plant material and growth conditions

Seeds of the rice (*Oryza sativa*) cultivar Nipponbare and *OsARP6*-RNAi transgenic lines were surface-sterilized and pre-germinated for 1 d at 37 °C followed by 2 d at 28 °C. Seeds were germinated under 12/12 h light/dark, 30/22 °C. At 2 weeks old, seedlings were transferred to a Yoshida Rice culture modified solution in a hydroponic system (Yoshida *et al.*, 1971; Secco *et al.*, 2013). The solution was renewed every 7 d. After 3 weeks, half of the seedlings were transferred to a solution without NaH_2_PO_4_ for a 24-h Pi-deficiency treatment.

### Development of *OsARP6*-RNAi plants

*OsARP6*-RNAi lines of rice (cv. Nipponbare) were made with the vector pFGC1008 (ABRC, https://abrc.osu.edu/), which targeted 489 bp within exons 2–7 of *OsARP6* (*LOC_Os01g16414*) and was mobilized into *Agrobacterium tumefaciens*. Multiple lines showed varying degrees of knockdown of *OsARP6*, and line #19 was selected for ChIP-Seq and RNA-Seq analysis.

### Transcript quantification by RT-qPCR

RNA was extracted from 0.1 g frozen shoots of wild-type and *OsOsARP6*-RNAi plants using the RNeasy Plant Mini kit (Qiagen) and was DNase-treated using RNase-Free DNase (Qiagen). cDNA was prepared from 1 ug total RNA using an oligo(dT) primer by the SuperScript III synthesis kit (Invitrogen) according to the manufacturer’s instructions. RT-qPCR was performed with a ViiA7 real-time PCR system using SYBR Green detection chemistry (Applied Biosystems) and gene-specific primers (IDT). Expression data were normalized to the expression level of the *OsActin* gene.

### Physiological and morphological measurements

Wild-type and transgenic lines of *OsARP6*-RNAi were used for physiological studies carried out as described by Joshi *et al.* (2014) under control conditions or after 7 d of Pi deficiency. SPSS-17 software (SPSS, Chicago, Illinois, USA) was used for statistical analyses. Means were compared by Duncan’s multiple range tests (DMRT) at the *P*<0.05 level. Student’s *t*-test was used to analyse the morphological parameters.

### OsH2A.Z antibody

Based on amino acid identity among the three rice H2A.Z sequences at the N terminus (Deal *et al.*, 2007), the peptide sequence N-AGKGGKGLLAAKTTAAK-C was synthesized as a four-fold multiple antigenic peptide (ProteinTech Group, Chicago, IL). Polyclonal antibodies were raised that reacted with the relatively conserved N termini of the rice H2A.Z histone variant subclass proteins HTA705, HTA712, and HTA713, but not with representatives of the other three H2A subclasses. The primary injection and three subsequent boosts were done with 250 mg of peptide in rabbit. Immunoblotting of recombinantly expressed HTA713 was used for antibody validation (see Supplementary Dataset 6 at *JXB* online).

### ChIP-Seq

The chromatin immunoprecipitation (ChIP) experiments were carried out as described previously (Smith *et al.*, 2010; Widiez *et al.*, 2014) with minor modifications (Supplementary Dataset 6) on rice seedlings grown under 24-h Pi deficiency or control conditions. The ChIP DNA was cleaned using a DNA Clean & Concentrator kit (Zymo Research). ChIP-Seq libraries were prepared with the Hyper Library Construction Kit from Kapa Biosystems with two modifications: adaptors were diluted 1:20 and DNA was amplified for 10 cycles. Libraries were quantitated by qPCR and sequenced for 101 cycles from one end of the fragments on a HiSeq2500 using a HiSeq SBS sequencing kit version 4. Fastq files were generated and demultiplexed with the bcl2fastq v2.17.1.14 Conversion Software (Illumina). Illumina reads of all samples have been submitted to the Sequence Read Archive at the National Center for Biotechnology Information (NCBI, http://www.ncbi.nlm.nih.gov/sra) under accession number SRP102661. Sequenced reads were quality-checked using FastQC software (http://www.bioinformatics.babraham.ac.uk/projects/fastqc). Bowtie was used to uniquely align the reads to the reference genome (MSU Rice Genome Annotation Release 7.1) with up to two mismatches allowed (Langmead, 2010). Regions of H2A.Z enrichment were defined using the SICER software package (Zang *et al.*, 2009), with the input genomic DNA as a background control and pre-immune serum ChIP DNA as a negative control (parameters: W=200; G=200; FDR<0.01). Differential enrichment of H2A.Z was determined using SICER-df.sh shell script (parameters: W=200; G=200; FDR<0.01). After the positions of the peaks were determined, genes (including the 250 bp upstream and downstream) overlapping the peaks were considered to have H2A.Z enrichment using custom Perl scripts (Dassanayake *et al.*, 2009). In addition, ngs.plot was used to visualize the genome-wide enrichment pattern of H2A.Z through gene body regions using the reference genome (MSU Rice Genome Annotation Release 7.1). The k-means program in ngs.plot was used to divide the protein coding genes (PCGs) into three groups based on H2A.Z deposition patterns (k_1_–k_3_) (Shen *et al.*, 2014). To apply the gene ontology (GO) analysis, AgriGO was used with singular enrichment analysis (SEA) with the hypergeometric statistical test method with *P*≤0.05 (Du *et al.*, 2010).

### RNA-Seq

RNA was extracted from 0.1 g of frozen aerial parts of rice seedlings grown under 24-h Pi deficiency or control conditions using a RNeasy Plant Mini kit (Qiagen). The RNA-Seq libraries were prepared with a TruSeq Stranded mRNAseq Sample Prep kit (Illumina). The libraries were quantitated by qPCR and sequenced for 101 cycles from one end of the fragments on a HiSeq2500 using a HiSeq SBS sequencing kit version 4. Fastq files were generated and demultiplexed with the bcl2fastq v2.17.1.14 Conversion Software (Illumina). Illumina reads of all samples have been submitted to the Sequence Read Archive at the NCBI under accession number SRP102661. RNA-Seq reads for each sample were mapped to the reference genome (MSU Rice Genome Annotation Release 7.1) using the Bowtie2 tool (Langmead and Salzberg, 2012). To quantify transcript abundance, the Cuffdiff tool was applied to obtain fragments per kilobase of transcript per million mapped reads (FPKM) (Trapnell *et al.*, 2012). Differentially expressed genes were identified using the DESeq2 package (FDR<0.001) (Love *et al.*, 2014) and the normalized rLog (regularized Log-transformation) values of selected genes (*n*=2002) with FDR<0.001 obtained from DESeq2 were used for clustering. The fuzzy k-means clustering was done with the Aerie tool (Gasch and Eisen, 2002).

## Results and discussion

### H2A.Z is enriched at rice protein-coding genes and is linked to gene expression

The primary aim of this study was to examine the separate and combined impacts of a major environmental stress and knockdown of a key component of the SWR1 H2A.Z exchange complex on H2A.Z localization. We first examined global H2A.Z distribution by carrying out ChIP-Seq experiments on shoots of 36-d-old rice plants. Two biological replicates were examined to ensure reproducibility (Pearson correlation coefficient *r*=0.89; Supplementary Fig. S1A, Supplementary Table S1). All genes were separated into four categories according to their MSU7 annotation (Kawahara *et al.*, 2013; Zhang *et al.*, 2018): protein-coding genes (PCGs), pseudogenes (PGs, i.e. genes that are neither expressed nor transposable elements), transposable elements (TEs), and transposable element-related genes (TEGs). As shown in Supplementary Fig. S2, H2A.Z abundance varied among gene type, and was the highest and lowest for PCGs and TEs, respectively. These results were consistent with previous examinations of H2A.Z genic localization in rice (Zhang *et al.*, 2017), Arabidopsis (Zilberman *et al.*, 2008), and other model systems (Santisteban *et al.*, 2000; Adam *et al.*, 2001; Guillemette *et al.*, 2005; Zhang *et al.*, 2005; Whittle *et al.*, 2008; Tolstorukov *et al.*, 2009; Nekrasov *et al.*, 2012), demonstrating that H2A.Z deposition patterns are largely conserved among eukaryotes.

**Fig. 1.**
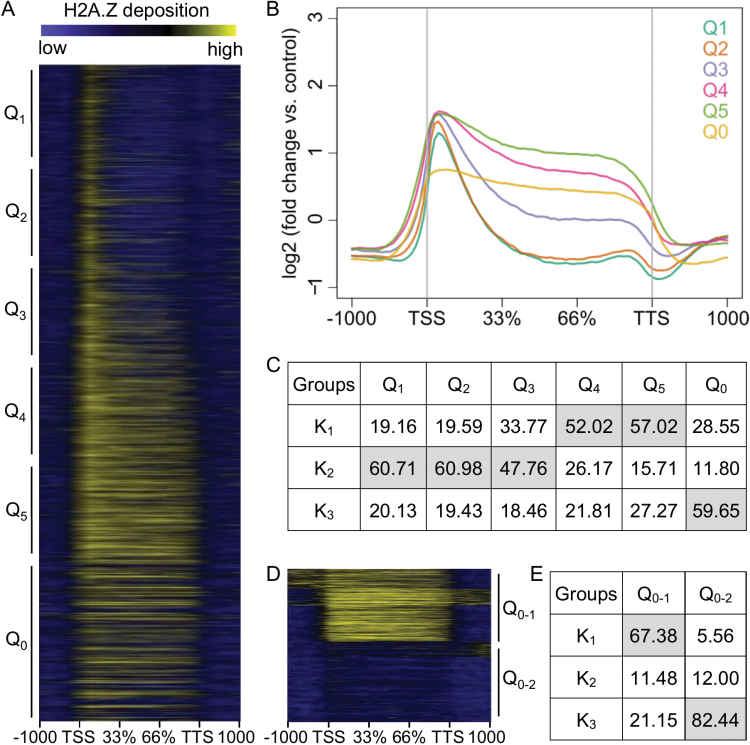
H2A.Z deposition is correlated with gene expression. (A) Heat map and (B) average profiles of H2A.Z deposition ranked according to expression in the wild-type under control conditions from 1000 bp upstream of the transcription start site (TSS) to 1000 bp downstream of the transcription termination site (TTS). Q_1_ is the highest expressed quintile and Q_5_ is the lowest expressed quintile. Q_0_ contains the genes with no expression (FPKM=0). (C) The correlation between quintiles with H2A.Z deposition patterns (k_1–3_). The numbers represent the normalized distribution of genes in each pattern. (D) Two distinct patterns of H2A.Z deposition for genes with no expression and (E) their correlation with H2A.Z deposition patterns (k_1–3_).

Studies in Arabidopsis and rice have shown a parabolic relationship between transcript abundance and H2A.Z deposition (Zilberman *et al.*, 2008; Coleman-Derr and Zilberman, 2012; Yelagandula *et al.*, 2014; Zhang *et al.*, 2017; Dai *et al.*, 2017). To further investigate this, we carried out RNA-Seq experiments on the same tissues used for ChIP-Seq (Supplementary Fig. S3, Fig. 1). The highest expressed genes in quintile 1 (Q_1_) and Q_2_ had a prominent H2A.Z peak near the TSS and a lesser peak near the transcription termination site (TTS). Reciprocally, low-expressed genes in Q_4_ and Q_5_ exhibited relatively higher H2A.Z deposition, particularly in the gene body (GB). Interestingly, genes in Q_0_ (i.e. not expressed) could be placed in two divergent sub-groups (Fig. 1D). Together, these results showed a general negative correlation between gene expression and H2A.Z enrichment, except for genes that had severe depletion of H2A.Z, and further demonstrate a parabolic relationship between H2A.Z abundance and gene expression in plants (Coleman-Derr and Zilberman, 2012; Dai *et al.*, 2017; Yelagandula *et al.*, 2014; Zhang *et al.*, 2017; Zilberman *et al.*, 2008).

### H2A.Z enrichment in rice PCGs exhibits three distinct deposition patterns

**Fig. 2.**
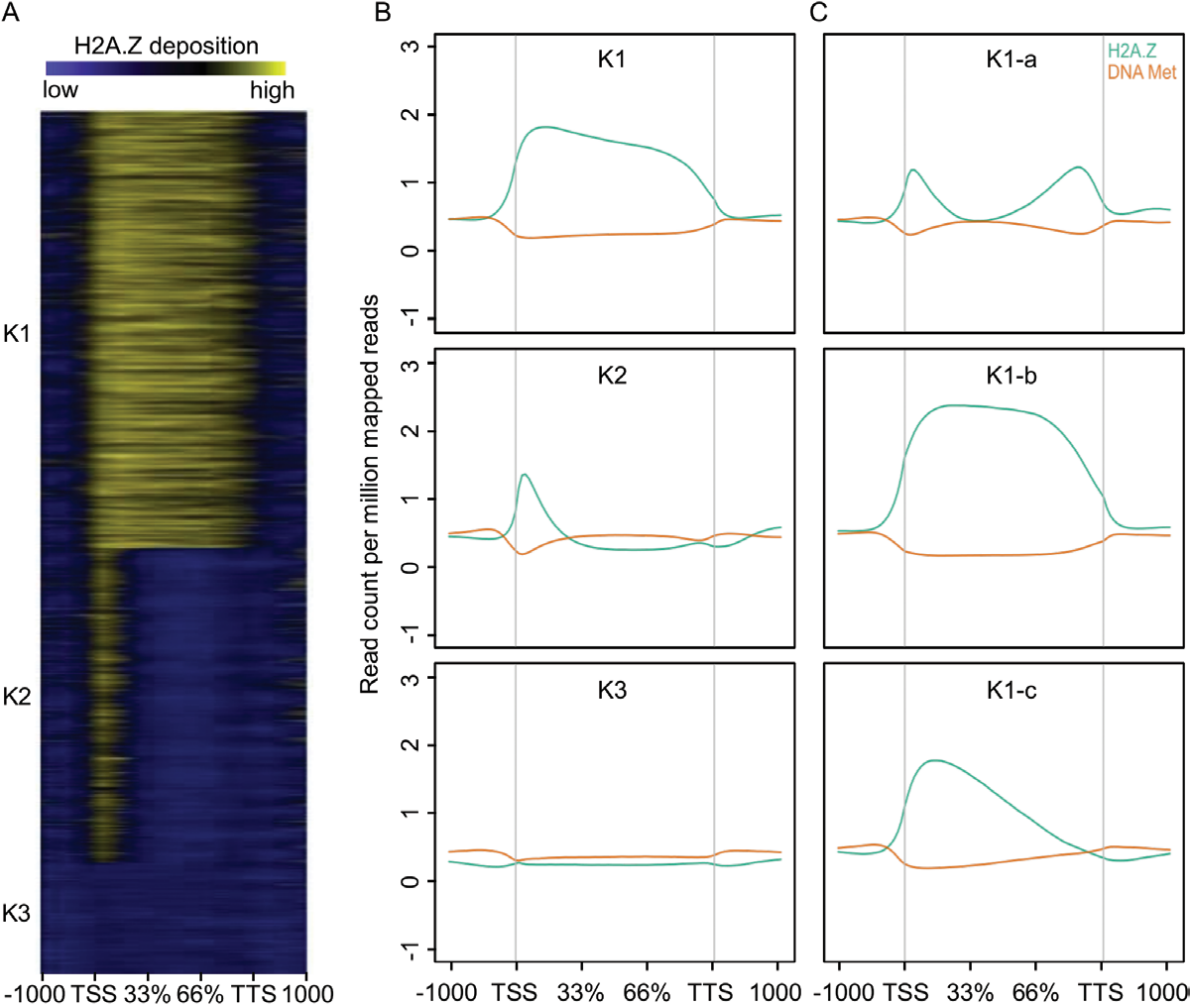
H2A.Z deposition patterns in rice protein-coding genes. (A) Heatmap and (B) average profiles of three distinct H2A.Z deposition patterns (k1–3) from 1000 bp upstream of the transcription start site (TSS) to 1000 bp downstream of the transcription termination site (TTS) with their average profiles of DNA methylation. The ChIP-Seq reads were normalized to control input reads. (C) The average profiles of three K1-subgroups with their average profiles of DNA methylation. The ChIP-Seq reads were normalized to control input reads.

A previous study in Arabidopsis indicated a correlation between H2A.Z distribution and gene responsiveness (Coleman-Derr and Zilberman, 2012). To investigate this in rice, we first sought distinct H2A.Z patterns among PCGs (Fig. 2). k-means clustering revealed three divergent patterns of H2A.Z deposition: two major groups (k_1_, *n*=18232 and k_2_, *n*=13128) and a minor group (k_3_, *n*=4740). In k_1_, H2A.Z distribution showed broad peaks inside the gene body (GB) with three distinct sub-patterns (Fig. 2C). Conversely, in k_2_, H2A.Z deposition was limited to a sharp peak near the TSS. Finally, in k_3_, an explicit depletion of H2A.Z was observed. To support the demarcation of genes into these three groups, we examined DNA methylation patterns, which have been shown to be antagonistic to H2A.Z abundance in Arabidopsis (Zilberman *et al.*, 2008) and rice (Zhang *et al.*, 2017). Using data from a recent study in rice (Secco *et al.*, 2015), we generated DNA methylation profiles of our k clusters. For each cluster, the DNA methylation pattern was inverse to that of H2A.Z abundance, particularly for the k_1_ sub-groups (Fig. 2B, C).

Comparison with gene expression showed that genes in the k_1_, k_2_, and k_3_ clusters tended to be low-expressed, high-expressed, and not expressed, respectively (Fig. 1C). GO enrichment analysis (Supplementary Dataset 1) with AgriGO (Du *et al.*, 2010) showed that k_1_ genes were significantly (FDR<0.05) enriched with transcription factor genes and genes related to secondary metabolism and stress-responses, whereas k_2_ genes were over-represented in primary metabolism and biosynthesis GO terms. Genes in the smaller k_3_ cluster comprised a combination of GO terms enriched in k_1_ and k_2_. Based on these results, we assigned the k_1_ genes as ‘stress-related’ and the k_2_ genes as ‘housekeeping’. Consistent with this, 82% of a group of rice genes (*n*=4147) considered as housekeeping genes in a previous study (Chandran *et al.*, 2016) were among the genes in our k_2_ cluster. Our data indicated correlations between a sharp peak of H2A.Z deposition at the TSS with housekeeping genes, and broad H2A.Z deposition in the GB of stress-related genes. This is similar to observations in Arabidopsis (Coleman-Derr and Zilberman, 2012), and suggests that H2A.Z deposition patterns are generally predictive of gene function in plants.

### Pi deficiency leads to a redistribution of H2A.Z across rice genes

Previously, we found enrichment of H2A.Z at a number of Pi deficiency-induced genes in Arabidopsis, which was lost upon Pi deficiency (Smith *et al.*, 2010). This suggested gene repression by H2A.Z under non-inductive conditions. Consistently, H2A.Z abundance at these loci was lower in *arp6* mutants, which led to increased transcript abundance despite the mutant plants being grown under Pi-sufficient conditions (Smith *et al.*, 2010). Whether this phenomenon occurs genome-wide or in other species has not been reported.

Numerous genes are differentially expressed in response to Pi deficiency in a short- or long-term manner (Thibaud *et al.*, 2010; Cai *et al.*, 2013; Secco *et al.*, 2013; Smith *et al.*, 2015). A systemic signaling network modulates ‘long-term’ genes, which are relatively specific to Pi and are differentially regulated one or more days following Pi deficiency. Signals from the shoot play an important role in this signaling network. Therefore, we sought to investigate the chromatin structure of nuclei in shoots in response to a 24-h Pi-deficiency treatment, during the relatively early stages of the long-term, systemic transcriptional response.

**Fig. 3.**
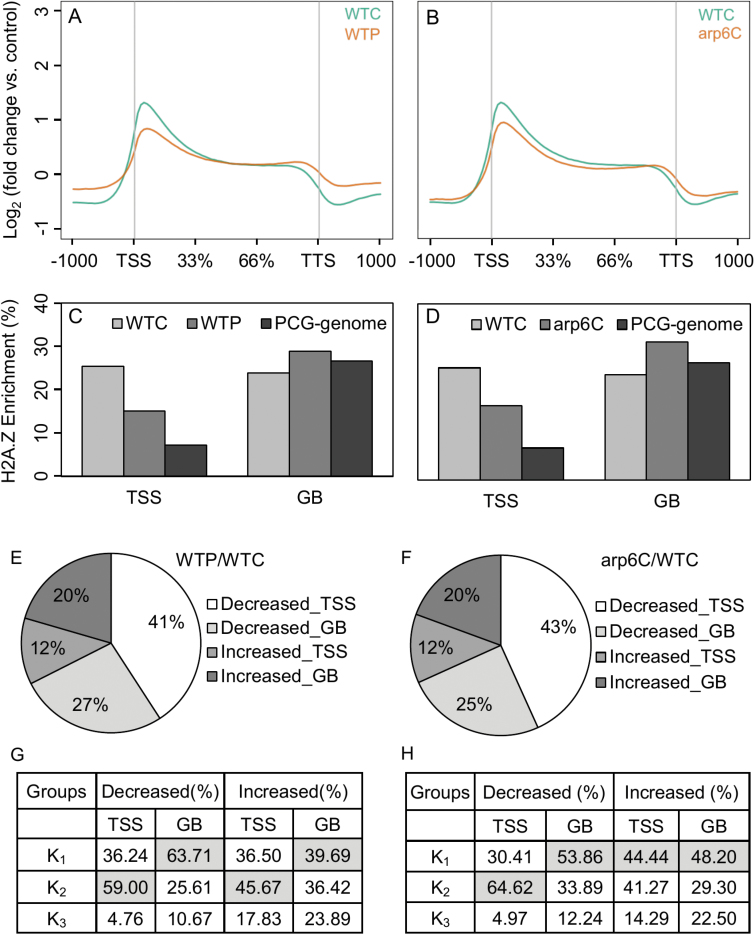
Differential H2A.Z enrichment across rice genes in response to Pi deficiency or *OsARP6*-knockdown. (A) Average profile of H2A.Z deposition in wild-type under control (WTC) and 24-h Pi deficiency (WTP), and (B) in WTC and *OsARP6*-RNAi (arp6C) under control conditions. (C) The percentage of H2A.Z distribution in different part of genes in WTC and WTP, and the related differential in protein-coding genes (PCG-Genome), and (D) in WTC and *OsARP6*-RNAi (arp6C) under control conditions and the related differential in PCG-Genome. (E) Distribution of differential H2A.Z enrichment in distinct part of genes in WTC and WTP, and (F) in WTC and arp6C under control conditions. (G) Normalized distribution of genes with differential H2A.Z enrichment in the K_1–3_ groups in WTC and WTP, and (H) in WTC and arp6C under control conditions.

In parallel with the experiments described above, we carried out ChIP-Seq experiments on shoots from Pi-deficient plants and examined the impact on H2A.Z distribution (two replicates, Pearson correlation coefficient *r*=0.99; Supplementary Fig. S1B, Table S1). To facilitate our analyses, we divided genes into a specific TSS proximal region (‘TSS’, 250 bp upstream to 500 bp downstream of the TSS) and gene body region (‘GB’, 500 bp downstream of the TSS to 250 bp downstream of the TTS). As shown in Fig. 3, there was a reduction in H2A.Z deposition at the TSS region in Pi-deficient wild-type (WTP) compared to the control wild-type (WTC), whereas an increase in H2A.Z deposition was observed in the GB. These results reflect an apparent redistribution of H2A.Z deposition from the TSS to the GB in response to Pi deficiency.

Next, we identified changes in H2A.Z deposition at specific genes. A total of 13989 PCGs contained at least one differential H2A.Z peak between the WTP and WTC samples. Approximately 68% contained a decrease in one or more H2A.Z peaks in WTP relative to WTC, whereas 32% contained an increase in one or more H2A.Z peaks. The majority (67%) of genes containing a decrease in H2A.Z in WTP exhibited the decrease in the TSS region (Fig. 3E). These genes were enriched in our k_2_ group (Fig. 3G), and showed over-representation of translation-related genes (Supplementary Dataset 2). Genes with a decrease in H2A.Z in the GB were enriched in cluster k_1_ (Fig. 3G) and were over-represented with transcription factor families. Of the genes with a gain of H2A.Z deposition in WTP, 20% contained the increased H2A.Z peak in the GB (Fig. 3E). These genes were more evenly distributed among our k clusters, having the highest proportion of k_3_ genes (Fig. 3G), and were enriched with protein kinases, particularly receptor-like kinases (Supplementary Dataset 2). The smallest proportion of genes containing a differential H2A.Z peak were those containing an increase at the TSS. These genes were somewhat enriched in cluster k_2_ (Fig. 3G), but yielded only one significantly enriched GO term (plasma membrane). Together, these data demonstrated that the major impacts of Pi deficiency on H2A.Z deposition were a reduction at the TSS that correlated with housekeeping genes (e.g. translation-related genes), a reduction at the GB that correlated with responsive transcription factor genes, and an increase in H2A.Z deposition in the GB, which correlated with receptor-like kinase genes.

### Pi deficiency-induced changes in H2A.Z abundance are correlated with differential gene expression

To explore correlations between changes in H2A.Z deposition and gene expression in response to Pi deficiency, we carried out RNA-Seq experiments on the same tissues used for ChIP-Seq (Supplementary Fig. S3). We identified 1548 differentially expressed genes (DEGs) in response to Pi deficiency: 805 genes were up-regulated and 743 down-regulated. Response to stimuli and stress were the top significantly enriched GO terms for the up-regulated DEGs, which contained a number of stress-induced transcription factors, cellular detoxification components, and receptor-like kinases. Down-regulated DEGs were enriched in genes linked to growth and synthesis of lipids and cell walls (Supplementary Dataset 2). These DEGs were in line with other Pi-deficient transcriptome studies in plants (Thibaud *et al.*, 2010; Cai *et al.*, 2013; Secco *et al.*, 2013).

To examine the H2A.Z distribution at the DEGs, we first compared the H2A.Z profile of all up- or down-regulated DEGs with all expressed genes. The groups of DEGs, whether up- or down-regulated, exhibited higher H2A.Z deposition than all expressed genes under both control and Pi deficiency conditions (Supplementary Fig. S4A, B), consistent with a positive correlation between H2A.Z and gene responsiveness (Sura *et al.*, 2017). In up-regulated genes, a large reduction of H2A.Z through the whole gene was observed under Pi deficiency (Fig. S4A). This suggested that under control conditions H2A.Z deposition acted to repress transcription of these stress-responsive genes. In contrast, the down-regulated genes exhibited a smaller reduction in H2A.Z at the TSS under Pi deficiency and a marginal increase across the GB (Fig. S4B).

**Fig. 4.**
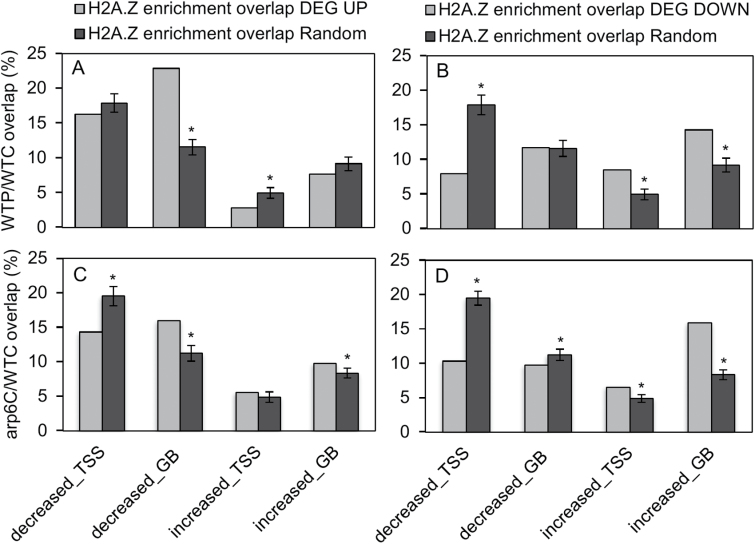
Bootstrapping analysis demonstrates correlations among genes containing a differential H2A.Z peak with (A) up-regulated and (B) down-regulated genes in the wild-type under 24-h Pi deficiency (WTP) compared to control conditions (WTC), or (C) up-regulated and (D) down-regulated genes in *OsARP6*-RNAi under control conditions (arp6C) compared to WTC. All data are means (±SD) for 1000 iterations. TSS, transcription start site; GB, gene body.

Next, we quantified the overlap between DEGs and genes containing a differential H2A.Z peak (Fig. 4). Compared to the same number of randomly selected genes, up-regulated DEGs were enriched among genes that contained a decrease in H2A.Z at the GB (1000 iterations, binomial test, *P*<0.001). Reciprocally, down-regulated DEGS were enriched among genes with an increase in H2A.Z at the GB (binomial test, *P*<0.001). These results revealed that differential expression in response to Pi deficiency was negatively correlated with H2A.Z levels in the GB. For example, genes encoding a WRKY transcription factor, glycosyl transferase, and receptor-like kinase, which showed up-regulation here as well as in 24-h Pi-deprived rice seedlings in a previous study (Cai *et al.*, 2013), exhibited a decrease in H2A.Z in their GB. On the other hand, genes encoding a kinesin, pectinesterase, and an enzyme implicated in lignin biosynthesis, (dihydroflavonol-4-reductase), showed down-regulation here and in the previous study (Cai *et al.*, 2013), and exhibited increases in H2A.Z in their GB. Our bootstrapping analyses also indicated a negative correlation between gene expression and H2A.Z levels at the TSS (Fig. 4A, B). Down-regulated DEGs were over-represented among genes with H2A.Z increases at the TSS and were under-represented among genes containing TSS H2A.Z decreases. In addition, up-regulated DEGs were under-represented among genes with H2A.Z increases at the TSS (binomial test, *P*<0.001). These observations are consistent with a repressive role for H2A.Z at the TSS, as was recently proposed in Arabidopsis (Dai *et al.*, 2017).

### Knockdown of *ARP6* in rice perturbs stress responses

The above results, together with previous work in Arabidopsis (Smith *et al.*, 2010; Kuo *et al.*, 2014), indicate a link between Pi deficiency responses and H2A.Z in plants. To further characterize the nature of this association, we sought to disrupt H2A.Z deposition in rice genetically and to combine this genetic perturbation with Pi deficiency. Because rice contains three H2A.Z-encoding genes (Deal *et al.*, 2007) we targeted a key component of SWR1c, ARP6, as a means to disrupt deposition of H2A.Z, as has been utilized in previous studies in Arabidopsis (Deal *et al.*, 2007; Kumar and Wigge, 2010; Smith *et al.*, 2010). Specifically, we generated *OsARP6*-knockdown lines via RNAi. Independent T3 generation *OsARP6*-RNAi lines showed varying degrees of knockdown of the *OsARP6* target locus (Supplementary Fig. S5), and three were selected for further analysis.

**Fig. 5.**
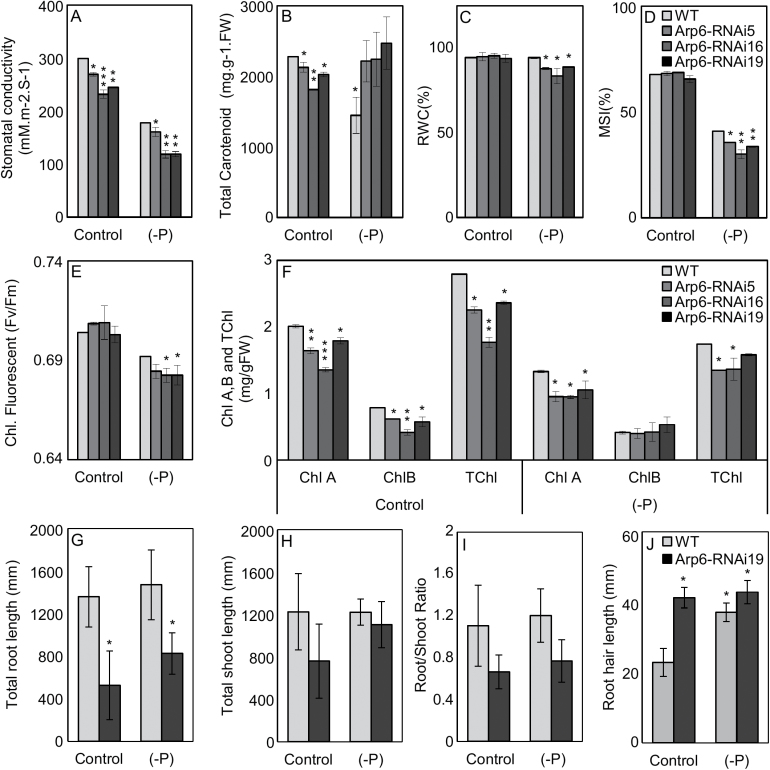
Assessment of physiological and morphological parameters in *OsARP6*-RNAi knockdown lines compared to the wild-type (WT) under control conditions and after 7 d of Pi deficiency. Stress indicators are (A) stomatal conductivity, (B) total carotenoid content, (C) relative water content (RWC), and (D) membrane stability index (MSI) (*n*=4). Quantifications of photosynthetic activity are (E) chlorophyll (Chl) fluorescence, (F) chlorophyll *a* (Chl A), chlorophyll *b* (Chl B), and total chlorophyll (TChl) (*n*=4). Significant differences compared to the WT control were determined using Duncan’s test: *P<0.05, **P<0.01, ***P<0.001. (G) Total root length, (H) total shoot length, and (I) root/shoot ratio (*n*=9). (J) Total root hair length (*n*=2). Significant differences compared to the WT control were determined using Student’s *t*-test: *P<0.05. All data are means (±SD).

We evaluated the *OsARP6*-RNAi lines for several stress indicators, including stomatal conductance, carotenoid levels, relative water content (RWC), and membrane stability index (Vijayalakshmi *et al.*, 2012; Havaux, 2014). Previous studies have typically shown marginal morpho-physiological responses to 24-h Pi-deficiency treatments (Secco *et al.*, 2013); therefore, we extended the duration of Pi deficiency for these experiments to 7 d. As shown in Fig. 5, all the *OsARP6*-RNAi lines exhibited decreases in stomatal conductance and carotenoids relative to the WT under control conditions. In response to Pi deficiency, stomatal conductance decreased in all genotypes, but remained significantly lower in the *OsARP6*-RNAi lines. Carotenoid levels also dropped in the WT in response to Pi deficiency, but remained at similar levels in the *OsARP6*-RNAi lines. RWC and membrane stability were similar among all genotypes under control conditions (Fig. 5C, D). During Pi deficiency, RWC was unchanged in the WT but decreased in the *OsARP6*-RNAi lines, whereas membrane stability decreased in all genotypes, but significantly more in the *OsARP6*-RNAi lines. Because photosynthetic activity is sensitive to many stressors, we next examined chlorophyll content and fluorescence (Fig. 5E, F). Under control conditions, a significant reduction was observed in chlorophyll *a*, chlorophyll *b*, and total chlorophyll in all *OsARP6*-RNAi lines compared to the WT (Duncan test, *P*<0.05; Fig. 5F). In response to Pi deficiency, chlorophyll levels decreased in all genotypes, and remained significantly lower in the *OsARP6*-RNAi lines relative to the WT except for the chlorophyll *b* content. Chlorophyll fluorescence was similar in all genotypes under control conditions, but showed a larger decrease in the *OsARP6*-RNAi lines in response to Pi deficiency (Fig. 5E). Together, these results indicated that knockdown of *OsARP6* elicited stress responses, which may have reflected up-regulation of stress-adaptive mechanisms during control conditions. The results also highlighted differences between the WT and *OsARP6*-RNAi lines in response to Pi deficiency, suggesting a link between OsARP6 and the modulation of Pi deficiency responses.

In Arabidopsis, *arp6* mutants display constitutive Pi starvation responses, including root phenotypes (Smith *et al.*, 2010). To test for a similar phenomenon in rice, we quantified several morphological parameters of *OsARP6*-RNAi plants under control conditions and in response to a 7-d Pi-deficiency treatment. Based on the physiological results and the *OsARP6* gene expression described above (Fig. 5, Supplementary S5A), the *OsARP6*-RNAi #19 line was selected for these analyses. Additional RT-qPCR experiments confirmed knockdown of the *OsARP6* target locus in *OsARP6*-RNAi #19 (Supplementary Fig. S5B). Under both control and Pi-deficiency conditions, *OsARP6*-RNAi had less root growth as compared to the WT (Fig. 5G). In contrast, shoot height and root:shoot ratios were similar between the WT and the *OsARP6*-RNAi line (Fig. 5H, I). For none of these parameters was there a difference in the genotypes with regard to Pi-deficiency response. As enhanced root hair proliferation is a common response to Pi deficiency (Nestler *et al.*, 2016), we next compared root hair length among the samples. As shown in Fig. 5J, the *OsARP6*-RNAi line exhibited longer root hairs than the WT under control conditions. In response to Pi deficiency, WT root hair length increased but was unchanged in *OsARP6*-RNAi. This is reminiscent of Arabidopsis *arp6* mutants, which exhibit constitutive Pi-deficiency responses, including increased root hair proliferation, despite growing under Pi-replete conditions (Smith *et al.*, 2010), and suggested an interaction between OsARP6-mediated H2A.Z deposition and Pi-deficiency responses in rice.

### The impact of *OsARP6*-knockdown on H2A.Z deposition is strikingly similar to that of Pi deficiency

To investigate the impact of *OsARP6*-knockdown on H2A.Z deposition, we first compared the average H2A.Z genic profiles of the WT and the *OsARP6*-RNAi #19 line (two replicates, Pearson correlation coefficient *r*=0.99; Supplementary Fig. S1C, Table S1). Surprisingly, the effect of *OsARP6*-knockdown was remarkably similar to that of Pi deficiency, namely an apparent redistribution of H2A.Z from the TSS to the GB (Fig. 3B, D). It has been widely accepted that loss of *ARP6* leads to dramatic reductions in deposition of H2A.Z in plants (Deal *et al.*, 2007; Kumar and Wigge, 2010; Smith *et al.*, 2010; Berriri *et al.*, 2016), despite the lack of clear reports of genome-wide localization of H2A.Z in mutants of SWR1c components (Zilberman *et al.*, 2008; Dai *et al.*, 2017). Because our *OsARP6*-RNAi plants maintained a low level of *OsARP6* expression (Supplementary Fig. S5A), it was possible that residual functionality of the SWR1c was maintained, leading to a relatively moderate decrease in H2A.Z abundance. However, in a recent study in Arabidopsis (Dai *et al.*, 2017), loss of *ARP6* resulted in a decrease in H2A.Z near the TSS, as shown in an average profile for all genes, but unexpectedly led to an increase in H2A.Z deposition at the 3′-end of a subset of genes, which is consistent with our findings. This suggests that our *OsARP6*-RNAi line effectively disrupted SWR1c-mediated H2A.Z deposition. Thus, the observed increases in H2A.Z abundance at the 3′-end of genes in the Arabidopsis *arp6* null mutant (Dai *et al.*, 2017) and our *OsARP6*-RNAi line may reflect an unbalanced influence on H2A.Z deposition by other mechanisms, possibly via the INO80 remodeling complex, which was recently implicated in 3′ genic deposition of H2A.Z in Arabidopsis (Zhang *et al.*, 2015). A total of 13707 PCGs showed differential H2AZ enrichment in *OsARP6*-RNAi compared to the WT, and the majority (70%) contained similar H2A.Z changes in response to Pi deficiency (Fig. 5B, D, F, H). Therefore, whether by Pi deficiency or knockdown of *OsARP6*, the generalized, major impacts on H2A.Z deposition were a reduction at the TSS of translation-related genes, a decrease in GB H2A.Z in responsive transcription factor genes, and an increase in GB H2A.Z deposition at receptor-like kinase genes (Supplementary Dataset 3).

Next, we sought to uncouple the impacts of Pi deficiency and *OsARP6*-knockdown on H2A.Z distribution by identifying genes containing differential H2A.Z peaks that were unique to either *OsARP6*-RNAi knockdown or Pi deficiency (Supplementary Fig. S6A). For genes containing a decrease in H2A.Z at the TSS, the *OsARP6*-RNAi unique group were enriched in GO terms related to plastid functions and translation, whereas those specific to Pi deficiency yielded no significantly enriched GO terms (Supplementary Dataset 4). On the other hand, for genes containing a decrease in H2A.Z in the GB, *OsARP6*-RNAi-specific genes yielded no enriched terms, but those unique to Pi deficiency were enriched in transcription factor and signaling-related genes. In addition, the *OsARP6*-RNAi unique genes with a decrease in H2A.Z were enriched in our k_2_ (housekeeping) gene group compared to the shared genes, whereas those unique to Pi deficiency were enriched in the k_1_ (stress-related) group (Supplementary Fig. S6B). For genes containing increases in H2A.Z deposition, whether at the TSS or GB, neither *OsARP6*-RNAi nor Pi-deficiency specific genes yielded enriched GO terms. Together, these results demonstrated that knockdown of *OsARP6* had a unique impact on loss of H2A.Z at the TSS relative to Pi deficiency, which affected housekeeping genes related to plastid functions and translation, whereas Pi deficiency led to a more biased impact on loss of H2A.Z at the GB, which affected stress-responsive transcription factor genes.

We next employed RNA-Seq on *OsARP6*-RNAi shoots (Supplementary Fig. S3) to identify DEGs between the WT and *OsARP6*-RNAi grown under control conditions. We identified 796 up-regulated and 1473 down-regulated DEGs in *OsARP6*-RNAi compared to the WT (FDR<0.001). Enriched GO categories for these DEGs were similar to those for DEGs in Arabidopsis *arp6* mutants (Berriri *et al.*, 2016; Sura *et al.*, 2017). Interestingly, the GO terms were also similar to those we obtained for Pi-deficient WT: response to stimulus and stress (e.g. WRKYs and receptor-like kinases) in up-regulated DEGs, and lipid, carbohydrate, and cell wall-related genes in down-regulated DEGs.

As with Pi-deficient WT (WTP), we identified correlations between *OsARP6*-RNAi DEGs and changes in H2A.Z deposition. Similar to WTP, *OsARP6* down-regulated DEGs were significantly over-represented for increases in H2A.Z in the GB, whereas up-regulated DEGs were over-represented for decreases in H2A.Z in the GB, revealing a negative correlation between expression and GB H2A.Z. In addition, down-regulated DEGs were over- and under-represented among genes containing increases and decreases, respectively, in H2A.Z at the TSS (binomial test, *P*<0.001). However, unlike WTP, whose up-regulated DEGs were under-represented among genes with increases in TSS H2A.Z, *OsARP6*-RNAi up-regulated genes were under-represented among genes containing decreases at the TSS. These results suggested that changes in H2A.Z at the TSS in response to Pi deficiency were reflective of H2A.Z having a repressive role, but that disruption of H2A.Z deposition in *OsARP6*-RNAi revealed a more complex role for H2A.Z at the TSS in which it could act to promote or repress transcription of different subsets of genes.

Similar to changes in H2A.Z, there was overlap between the DEGs in *OsARP6*-RNAi compared to WTP, but the overlap was less (Supplementary Fig. S6C). Of the 796 up-regulated genes in *OsARP6*-RNAi, 37% (*n*=297) were also up-regulated in WTP, and 41% (*n*=607) of the 1473 down-regulated genes were also down-regulated in WTP (Supplementary Fig. S7C). We next looked at significantly enriched GO terms for each group of unique genes. For up-regulated DEGs, those unique to *OsARP6*-RNAi were enriched in plastid and response-to-stimulus genes, whereas genes up-regulated only by Pi deficiency were enriched in response-to-stimulus (Supplementary Dataset 4). On the other hand, genes down-regulated uniquely in *OsARP6*-RNAi were enriched in plastid, carbohydrate metabolism, and cell wall GO terms, whereas those unique to Pi deficiency were not enriched in any terms (Supplementary Dataset 4). These results showed overlapping transcriptional changes in response to Pi deficiency and knockdown of *OsARP6*, but also revealed key differences. In particular, the GO term plastid was present in both sets of unique *OsARP6*-RNAi DEGs; those that were up-regulated tended to be genes also induced by a variety of stressors, such as lipoxygenases, whereas those down-regulated were linked to translation and chlorophyll synthesis.

### H2A.Z distribution in *OsARP6*-RNAi shoots does not change significantly in response to Pi deficiency

Our results indicated that Pi deficiency and *OsARP6*-RNAi each had a similar, profound impact on H2A.Z distribution. To examine the combined impact of these perturbations, we carried out ChIP-Seq on shoots of *OsARP6*-RNAi (arp6P) exposed to 24 h of Pi deficiency (two replicates, Pearson correlation coefficient *r*=0.99; Supplementary Fig. S1D; Table S1). As shown in Supplementary Fig. S7A, the genic H2A.Z profile for arp6P is very similar to that for WTP and arp6C. In contrast to the WT, *OsARP6*-RNAi exhibited many fewer genes with a gain or loss of H2A.Z (*n*=5202) in response to Pi deficiency (i.e. arp6C vs. arp6P). This suggested that knockdown of *OsARP6* or a 24-h Pi-deficiency treatment had dramatic but similar impacts on H2A.Z localization, and that the combined perturbations led to only a moderate additive disruption of H2A.Z localization. To test this possibility, we compared the overlap in differences in H2A.Z deposition between each of the ‘stress’ samples (WTP, arp6C, and arp6P) to WTC (Supplementary Fig. S7). For each combination there were very similar numbers of genes containing differential H2A.Z peaks, with an overlap of more than 60% (Fig. S7B). This confirmed that the impact on H2A.Z deposition in the arp6C, WTP, and arp6P samples was very similar and that the combined effects of *OsARP6*-RNAi knockdown and Pi deficiency did not result in a large additive perturbation on H2A.Z deposition. These results also raised the possibility that *OsARP6*-knockdown mimicked a Pi-deficient condition, which resulted in constitutive Pi deficiency responses under control conditions.

### Clustering analysis defines distinct interactions among transcriptional responses to Pi deficiency and *OsARP6*-knockdown

**Fig. 6.**
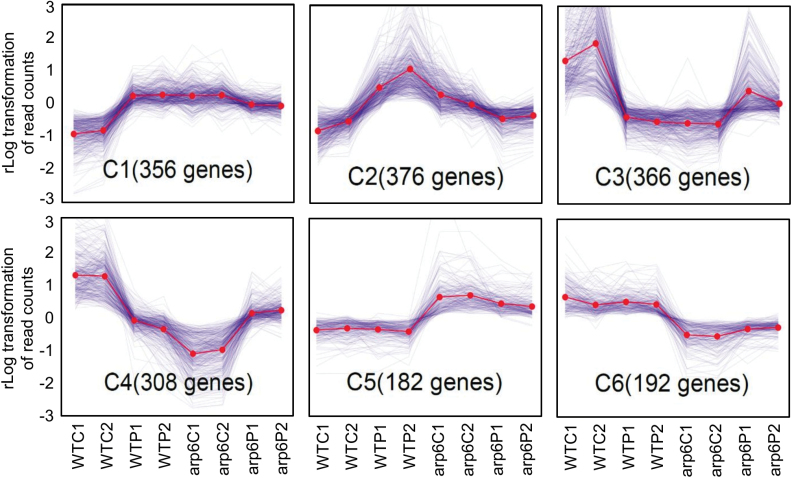
Six major clusters of 2002 protein-coding genes selected based on differential expression in response to Pi deficiency or *OsARP6*-knockdown or both (FDR<0.001). WTC1 and WTC2 are replicates 1 and 2 of the wild-type under control conditions. WTP1 and WTP2 are replicates 1 and 2 of the WT under Pi deficiency. arp6C1 and arp6C2 are replicates 1 and 2 of *OsARP6*-RNAi knockdown under control conditions. arp6P1 and arp6P2 are replicates 1 and 2 of *OsARP6*-RNAi under Pi deficiency.

To further investigate the interactions among *OsARP6*-knockdown and Pi deficiency, we employed clustering analysis in the deseq2 software package on RNA-Seq data for WTC, WTP, arp6C, and arp6P (Supplementary Fig. S8A). PCGs with significant differential expression (*n*=2002) were obtained for downstream clustering (FDR<0.001), which resulted in 14 distinct clusters. Most of the DEGs fell into one of six major clusters (C_1_–C_6_; Fig. 6). Genes in clusters C_1_ and C_2_ showed up-regulation in WTP and arp6C, and less so in arp6P, as compared to WTC. Enriched GO terms in these clusters were linked to stress responses and the plasma membrane, including many transporter genes such as two that encode Pht1 family Pi transporters (Supplementary Dataset 5). These clusters also contained several genes considered as ‘core plant PSR’ (phosphate starvation response) genes in rice (Secco *et al.*, 2013). In contrast to C_1_ and C_2_, genes in clusters C_3_ and C_4_ exhibited an opposite trend: down-regulation in WTP and arp6C, and to a lesser extent in arp6P, compared to WTC (Fig. 6). Genes from enriched GO terms in these clusters included those related to carbohydrate and lipid metabolism, secondary metabolism, and the extracellular region, including several expansins and kinesins (Supplementary Dataset 5). That the majority of DEGs comprised these four major clusters confirmed that *OsARP6*-knockdown and Pi deficiency elicited similar changes to the transcriptome, without a major synergistic effect. Many stress-responsive genes were up-regulated by either Pi deficiency or *OsARP6*-knockdown, whereas many metabolic and growth-related genes were down-regulated. The remaining two major clusters contained genes whose transcript abundance was altered in response to knockdown of *OsARP6* but not Pi deficiency. Cluster C_5_ genes were up-regulated in *OsARP6* compared to the WT regardless of Pi status (Fig. 6). Although no enriched GO terms were present in this cluster, the majority of the genes were in our k_1_ group (stress-related). Also of note in this cluster was the presence of three core plant PSR genes despite the lack of differential expression in response to Pi deficiency, which may have been due to an insufficient duration of Pi deficiency in this study. Cluster C_6_ genes exhibited an obvious down-regulation in arp6C and arp6P compared to WTC and WTP (Fig. 6), and these were enriched in our k_2_ group (housekeeping). Enriched GO terms in this cluster included plastid and thylakoid (Supplementary Dataset 5). Interestingly, 10 of these ‘plastid’ DEGs, which were implicated in several housekeeping functions in chloroplasts including chlorophyll biosynthesis, contained a decrease in H2A.Z at their TSS in arp6C and arp6P, but not WTP. These genes are examples of housekeeping genes that require H2A.Z deposition at their TSS for expression. As a result, knockdown of *OsARP6* led to decreased H2A.Z at the TSS and a corresponding reduction in transcript abundance. The specific reductions in transcription of the genes implicated in chlorophyll biosynthesis were consistent with the decreased chlorophyll content observed in *OsARP6*-RNAi plants (Fig. 5F).

In addition to the six major clusters described above, the interaction analysis resulted in a number of minor clusters (Supplementary Fig. S8B, Dataset 5). Two of these, C_14_ and C_9_, contained genes that were up- and down-regulated by Pi deficiency, respectively, but unaffected by *OsARP6*-knockdown. In contrast to the *OsARP6*-specific genes described above, these Pi-specific DEGs were fewer in number and were not enriched in any GO terms. Of the remaining clusters, three yielded enriched GO terms despite containing small numbers of genes. Genes in C_13_ were enriched in growth-related GO terms, which included a kinesin and several expansins. The transcript response of this cluster was similar to clusters C_3_ and C_4_, in which kinesins and expansins were also present. Finally, two clusters, C_11_ and C_12_, exhibited complex interactions between *OsARP6*-knockdown and Pi deficiency. Cluster C_11_ genes, which exhibited down-regulation by Pi in the WT but not in the *OsARP6*-RNAi line, were enriched in response to stimulus, and one gene was a core plant PSR gene. Genes in cluster C_12_ were up-regulated by both *OsARP6*-knockdown and Pi deficiency, and their expression was even higher in Pi-deficient *OsARP6*-RNAi shoots. These genes were enriched in transcription factor genes and highlighted a unique group of transcription factors that may respond to both Pi deficiency and *OsARP6*-knockdown perturbations, but via different mechanisms.

## Conclusions

Our results demonstrated that a 24-h Pi-deficiency treatment or knockdown of a SWR1c component had similar, profound effects on both the rice transcriptome and global genic distribution of H2A.Z. This suggests that these perturbations affected similar chromatin-remodeling and/or transcriptional regulatory mechanisms. Indeed, combining Pi deficiency with *OsARP6*-knockdown did not result in a significant additive effect. Because Pi deficiency is both an acute environmental stress and deprivation of a major essential nutrient, it elicits transcript changes in numerous housekeeping and stress-responsive genes. Our findings are consistent with a role for H2A.Z in the induction and/or maintenance of this transcriptional response. Accordingly, disruption of H2A.Z exchange via *OsARP6*-knockdown mimicked the onset of Pi-deficiency responses under control conditions. Although extensive overlap was observed for Pi deficiency and *OsARP6*-knockdown, there were also notable differences. Pi deficiency resulted in a biased impact on differential expression of stress-responsive genes that was negatively correlated with H2A.Z in the GB. In contrast, *OsARP6*-knockdown was biased toward differential gene expression of housekeeping genes linked to differential H2A.Z peaks at the TSS. Our results indicate that GB-localized H2A.Z plays a prominent role in repressing stress-responsive genes under non-inductive conditions. In contrast, the role of H2A.Z at the TSS appears to be more context-dependent in that it behaves as a repressor of particular stress-responsive genes, but acts to promote expression of some genes with housekeeping functions, such as those linked to translation and chloroplast functions. This may reflect a role for H2A.Z in promoting DNA accessibility, which can be followed by recruitment of active or repressive complexes according to target gene function.

## Supplementary data

Supplementary data are available at *JXB* online.

Table S1. Total reads, mapped reads, and peak counts for ChIP-Seq samples.

Fig. S1. Number of H2A.Z enrichment peaks in each replicate and the overlap between replicates.

Fig. S2. The average profiles of H2A.Z deposition among gene annotation types in wild-type shoots.

Fig. S3. PCA plot of RNA-Seq samples.

Fig. S4. The average H2A.Z deposition profiles of differentially expressed genes.

Fig. S5. Development of *OsARP6*-RNAi transgenic lines.

Fig. S6. Proportion of common and unique differential H2A.Z deposition genes.

Fig. S7. Average H2A.Z profiles and overlap between differential H2A.Z peaks and differentially expressed genes for all samples.

Fig. S8. Cluster analysis of differentially expressed genes.

Dataset 1. Significantly enriched GO terms for three distinct H2A.Z genic profile gene groups.

Dataset 2. Significantly enriched GO terms for genes with differential H2A.Z peaks or differential expression between wild-type grown under control and Pi-deficient conditions.

Dataset 3. Significantly enriched GO terms for genes with differential H2A.Z peaks or differential expression between *OsARP6*-RNAi and wild-type grown under control conditions.

Dataset 4. Significantly enriched GO terms for differential H2A.Z peaks and differentially expressed genes common or unique to WTC versus WTP or WTC versus arp6C.

Dataset 5. Significantly enriched GO terms for genes within clusters based on interactions among differentially expressed genes in WTC, WTP, arp6C, and arp6P.

Dataset 6. ChIP protocol and determination of OsH2A.Z antibody specificity.

## Supplementary Material

supplementary_Table_S1_figures_S1_S8

supplementary_dataset_S1

supplementary_dataset_S2

supplementary_dataset_S3

supplementary_dataset_S4

supplementary_dataset_S5

supplementary_dataset_S6
