# Supplementary material for: Rice H2A.Z negatively regulates genes responsive to nutrient starvation but promotes expression of key housekeeping genes
Source: J Exp Bot. 2018 Jun 28;69(20):4907–19. doi: 10.1093/jxb/ery244 (PMC6137989; doi:10.1093/jxb/ery244)
Supplement: supplementary_Table_S1_figures_S1_S8 [file ery244_suppl_supplementary_table_s1_figures_s1_s8.pdf]

Rice H2A.Z negatively regulates genes responsive to nutrient starvation but promotes expression of key housekeeping genes.

Supplementary Table S1

		H2AZ-Rep1	H2AZ-Rep2
**Arp6-RNAi (+P)**	Total Reads	46141651	28346243
	Mapped Reads (%)	40200252(87.12)	23721208(83.68)
	Number of Peaks	43430	40706
**Arp6-RNAi (-P)**	Total Reads	37739099	39813678
	Mapped Reads (%)	32281474(85.54)	34214172(85.94)
	Number of Peaks	43521	42796
**WT (+P)**	Total Reads	43352006	41931079
	Mapped Reads (%)	30135234(69.51)	35226201(84.01)
	Number of Peaks	42701	42422
**WT (-P)**	Total Reads	41925535	37529437
	Mapped Reads (%)	34681088(82.72)	30196530(80.46)
	Number of Peaks	43123	45016

Table S1. The number of total reads, mapped reads and H2A.Z enrichment peaks of *OsARP6*-RNAi under control (+P) and 24-hours P-deficiency (-P), WT under control (+P) and 24-hours P-deficiency (+P). Each sample contains two replicates.

Supplementary Figure S1

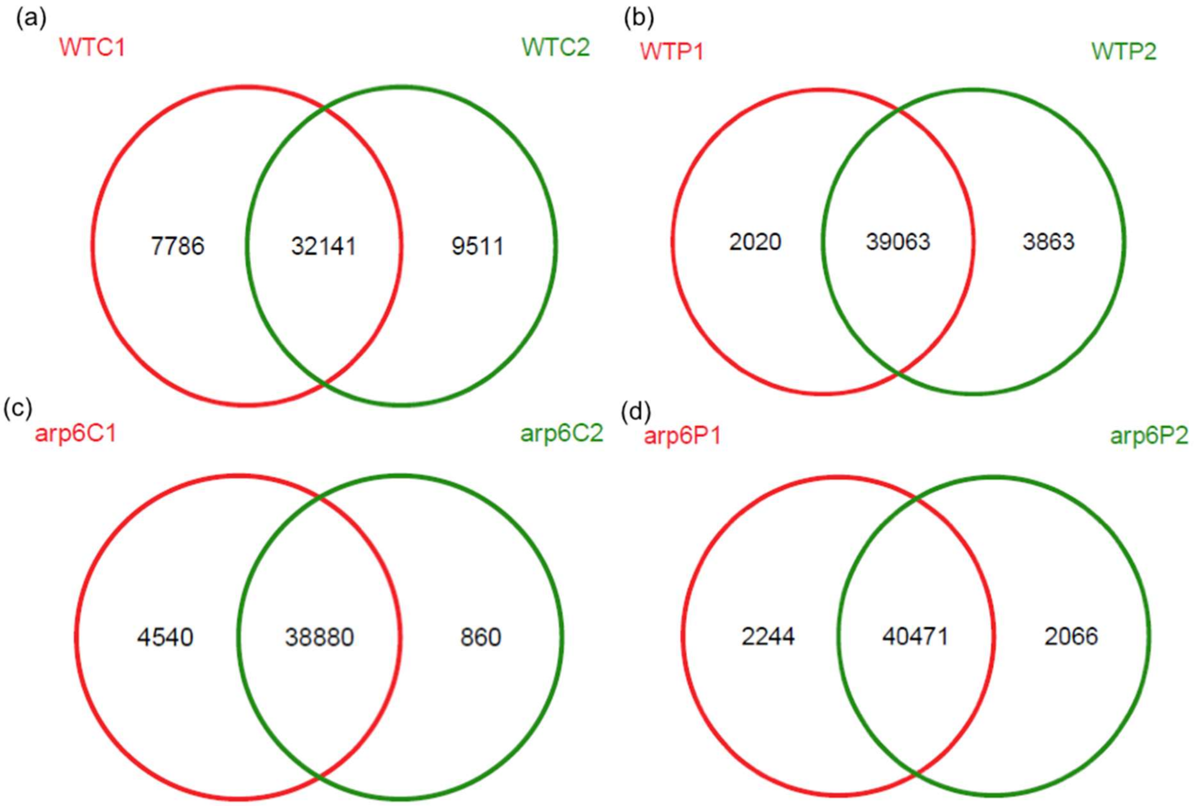

Figure S1. Number of H2A.Z enrichment peaks in each replicate and the overlap between two replicates in (a) WT under control (WTC) and (b) Pi-deficiency conditions (WTP) and (c) *OsARP6*-RNAi under control (arp6C) and (d) Pi-deficiency conditions (arp6P).

Supplementary Figure S2

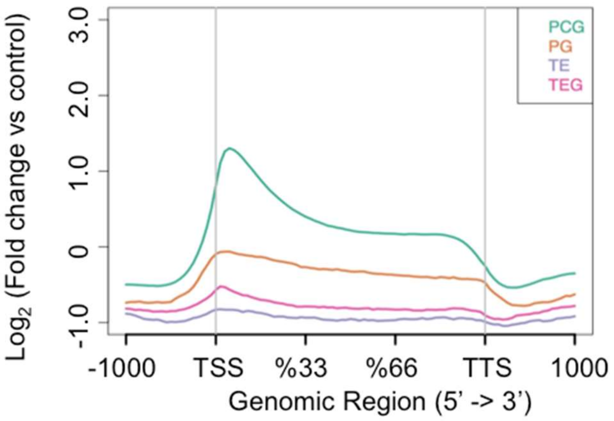

Figure S2. The average profile of H2A.Z deposition among the gene types in shoots from wild-type seedlings grown under control conditions (WTC). PCG: protein coding genes, PG: pseudogenes, TE: transposable elements, TEG: transposable element-related genes.

Supplementary Figure S3

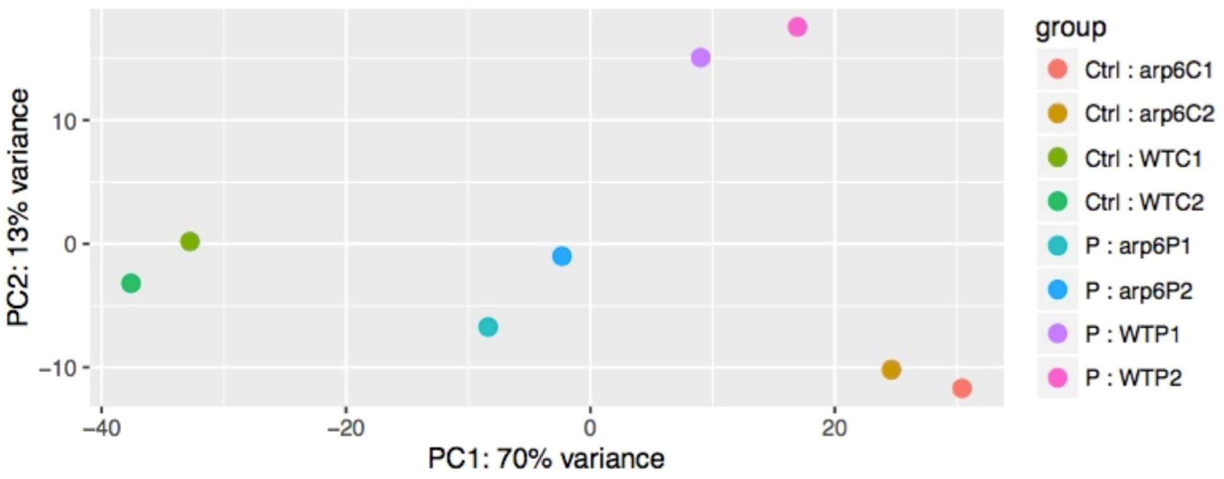

Figure S3. PCA plot of two replicates each of RNA-Seq of WT under control (WTC) and Pi deficiency (WTP) conditions, and *OsARP6*-RNAi under control (arp6C) and Pi deficiency (arp6P) conditions.

Supplementary Figure S4

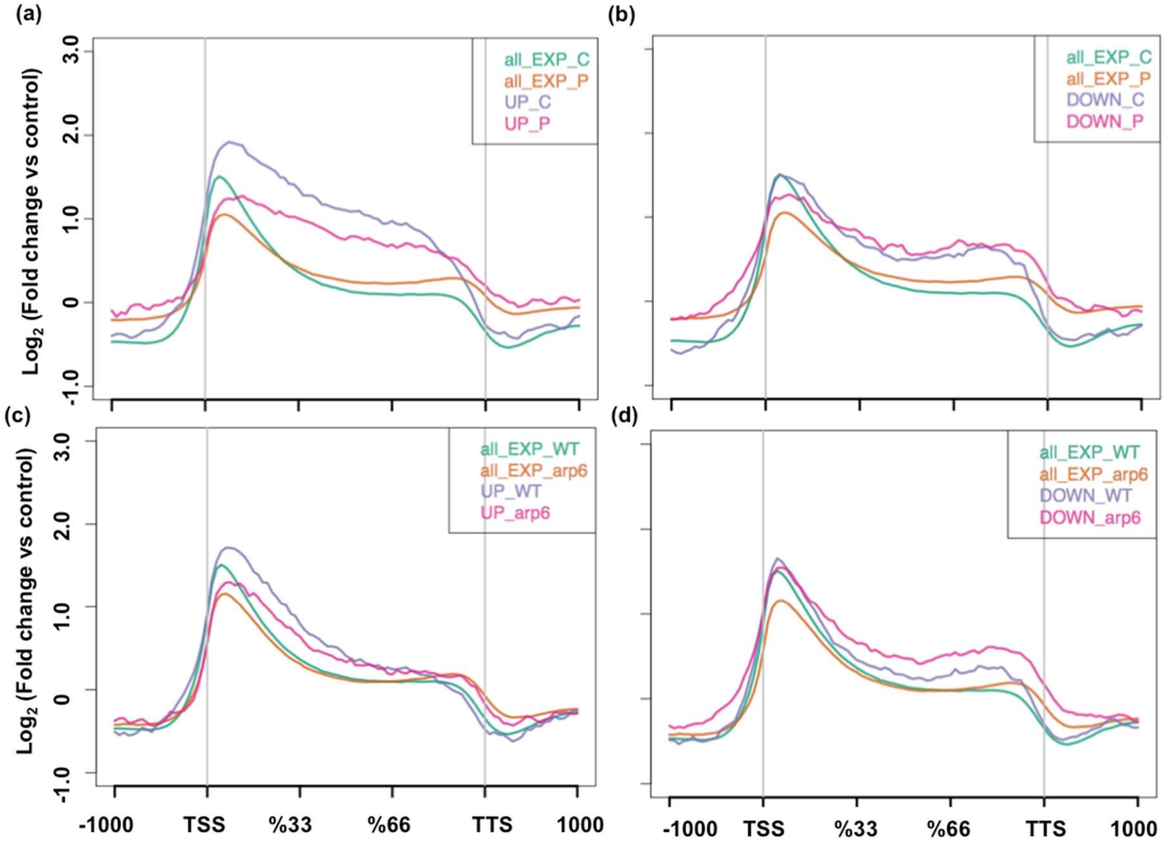

Figure S4. The average profile of H2A.Z deposition of up-regulated (a) and down-regulated (b) genes in WT under control and 24-hour Pi deficiency, and up-regulated (c) and down-regulated (d) genes in WT and *OsARP6*-RNAi (arp6) under control conditions. all_Exp: the average profile of H2A.Z deposition in “all expressed” genes, UP: the average profile of H2A.Z deposition of up-regulated genes, DOWN: the average profile of H2A.Z deposition of down-regulated genes.

Supplementary Figure S5

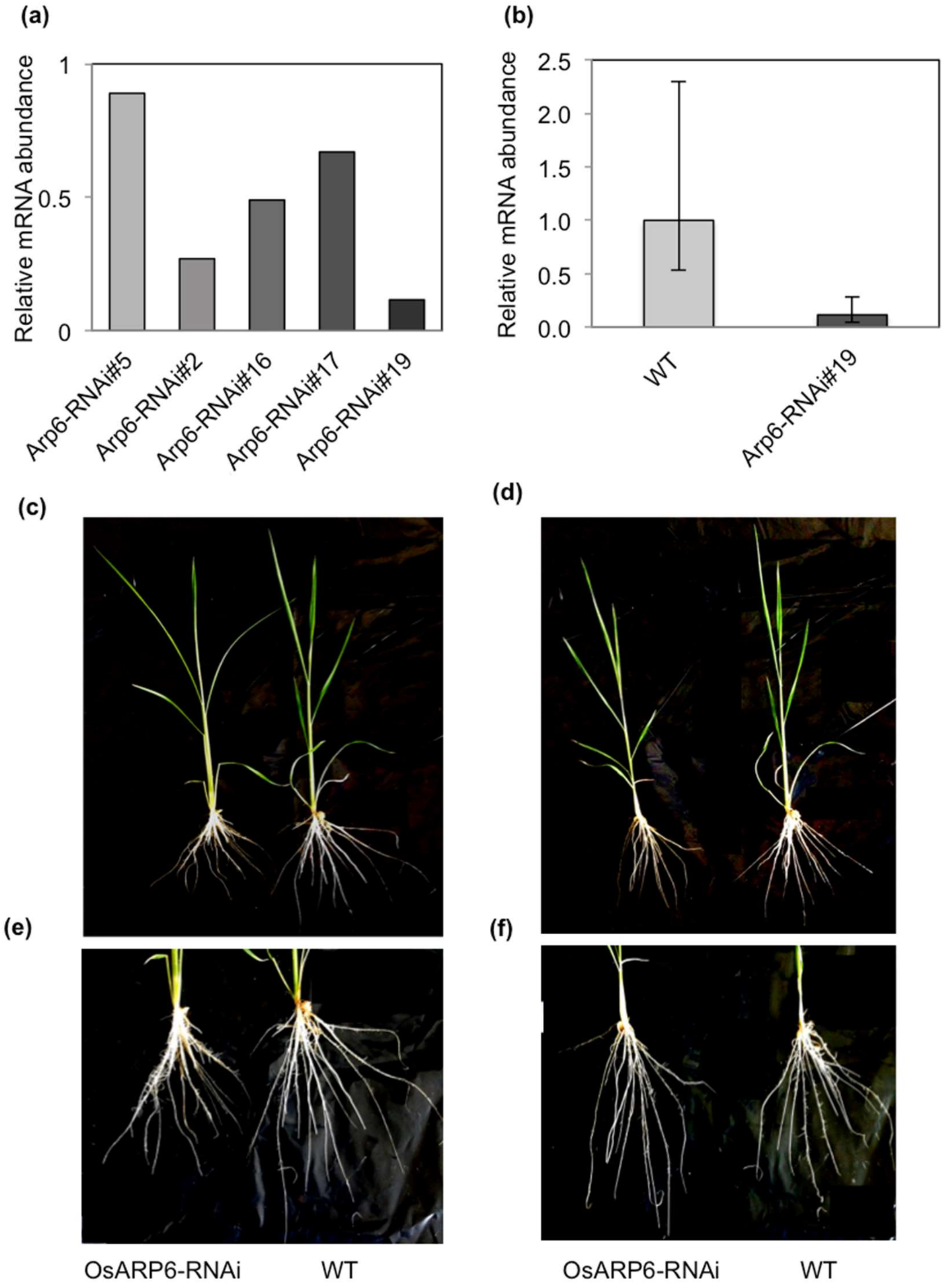

Figure S5. Development of *OsARP6*-RNAi Transgenics. (a-b) RT-qPCR results of *OsARP6*-RNAi lines with varying degrees of knockdown of the *OsARP6* target locus. (c-d) whole seedlings and (e-f) roots of *OsARP6*-RNAi knockdown compared to wild type (WT) grown under (c-e) control conditions and (d-f) Pi deficiency.

Supplementary Figure S6

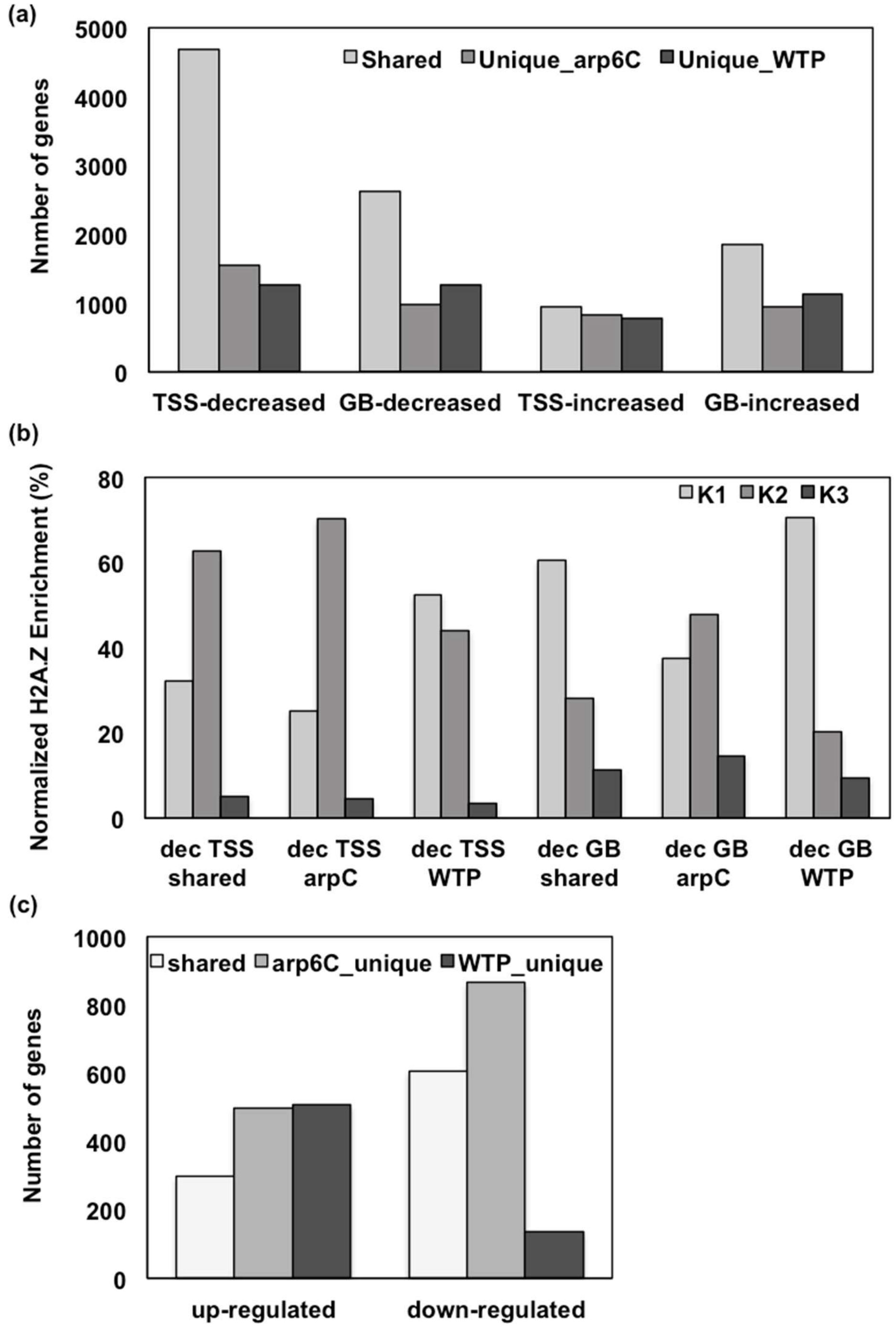

Figure S6. (a-b) Proportion of common and unique differential H2A.Z deposition genes in *OsARP6*-RNAi under control (arpC) and WT under 24-hour Pi deficiency (WTP) compared to WT under control conditions (WTC). (a) Number of common and unique genes. (b) Distribution ratio of common and unique genes in the K1-3 gene groups. (c) Proportion of common and unique differentially expressed genes in *OsARP6*-RNAi under control (arpC) and WT under 24-hour Pi deficiency (WTP) compared to WT under control conditions (WTC).

Supplementary Figure S7

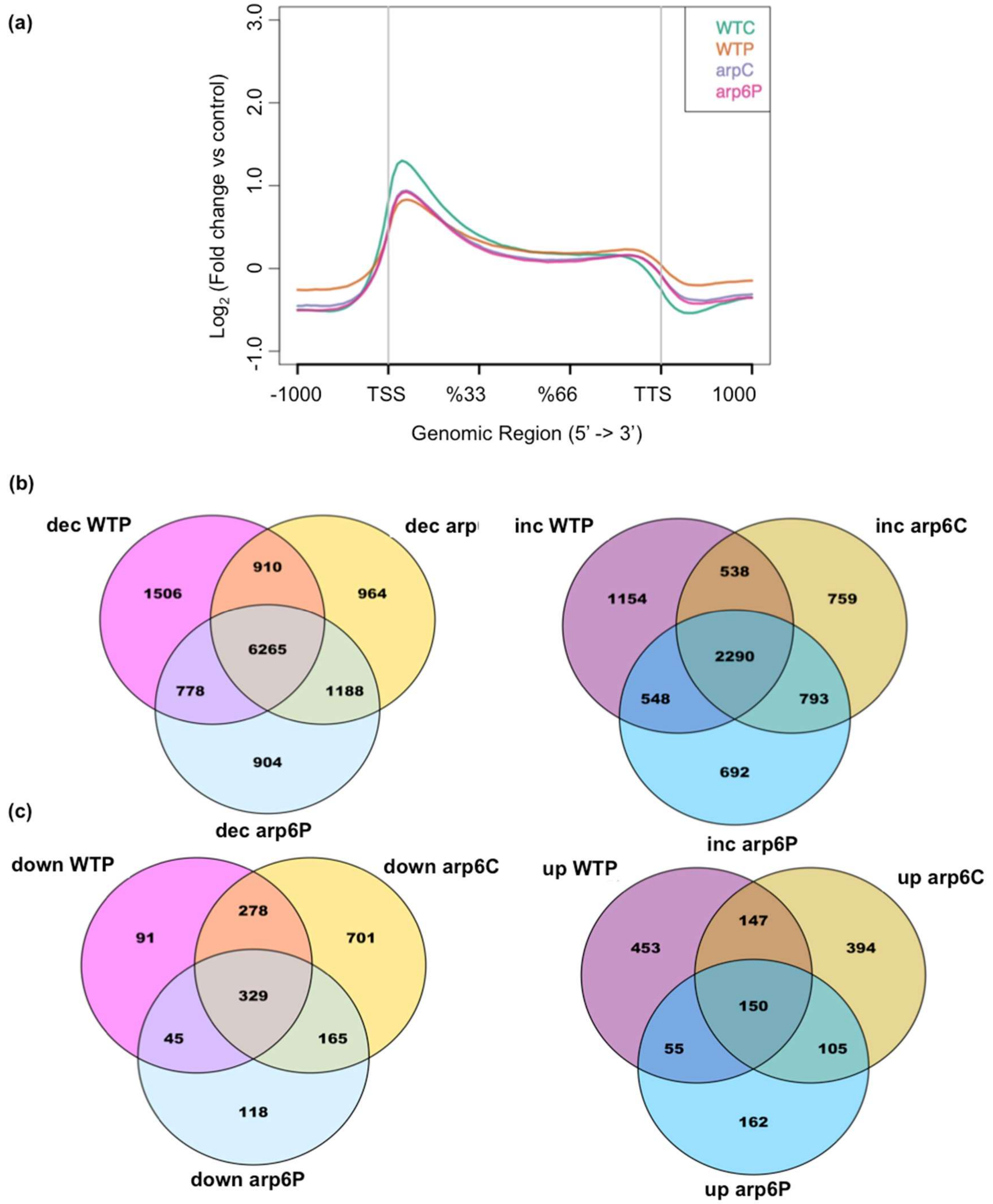

Figure S7. (a) Average profiles of H2A.Z deposition of WT under control (WTC) and Pi deficiency (WTP) conditions and *OsARP6*-RNAi line under control (arp6C) and Pi deficiency (arp6P) conditions. (b-c) The overlap of (a) differential H2A.Z deposition and (b) differentially expressed genes in WT under Pi deficiency (WTP), *OsARP6*-RNAi under control (arp6C) and Pi deficiency (arp6P) conditions compared to WT under control conditions (WTC).

Supplementary Figure S8

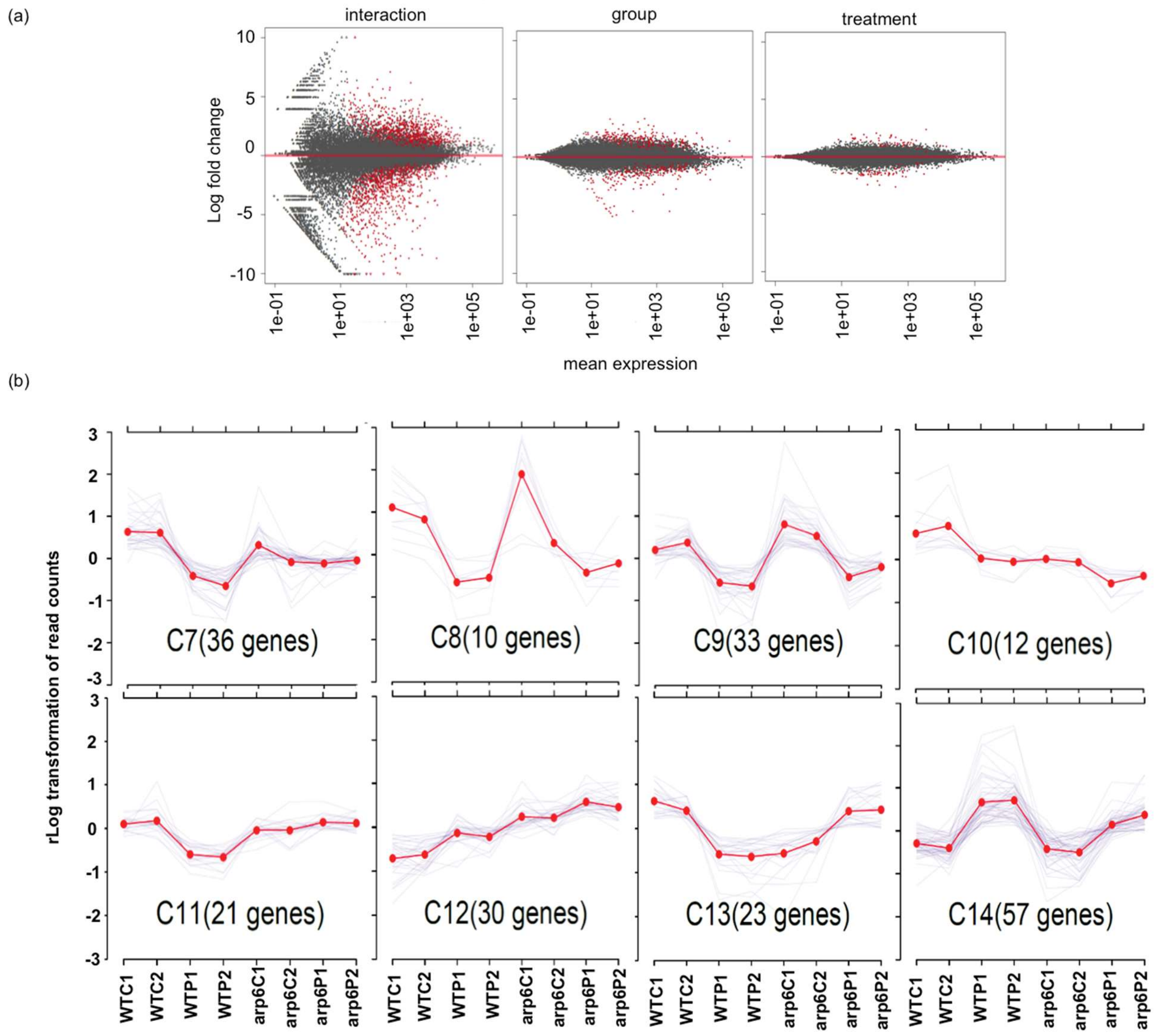

Figure S8. (a) Differentially expressed genes in both (interaction) or each of WT vs *OsARP6*-RNAi (group) under control vs Pi deficiency conditions (treatment) (FDR<1.00E-03). (b) Eight minor clusters of 2,002 PCGs selected based on differential expression in response to Pi deficiency or *OsARP6* knockdown or both (FDR<1.00E-03). WTC1 and WTC1, replicates 1 and 2 of WT under control conditions. WTP1 and WTP2, replicates 1 and 2 of WT under Pi deficiency. arp6C1 and arp6C2, replicates 1 and 2 of *OsARP6*-RNAi knockdown under control conditions. arp6P1 and arp6P2, replicates 1 and 2 of *OsARP6*-RNAi under Pi deficiency conditions.
