# Supplementary material for: Rice H2A.Z negatively regulates genes responsive to nutrient starvation but promotes expression of key housekeeping genes
Source: J Exp Bot. 2018 Jun 28;69(20):4907–19. doi: 10.1093/jxb/ery244 (PMC6137989; doi:10.1093/jxb/ery244)
Supplement: supplementary_dataset_S6 [file ery244_suppl_supplementary_dataset_s6.pdf]

Rice H2A.Z negatively regulates genes responsive to nutrient starvation but promotes expression of key housekeeping genes.

Supplementary dataset 6: Chromatin immunoprecipitation protocol and determination of OsH2A.Z antibody specificity

Chromatin Immunoprecipitation Protocol
(adapted from Smith et al., 2010 and Widiez et al., 2014)

Chromatin Immunoprecipitation Protocol PART I

1) Grind 1-4 g fresh or frozen tissue in liquid nitrogen.

2) Add 25 ml Nuclei Isolation Buffer (NIB)* and transfer the slurry into 50ml conical tube. Invert several times. Cross-link at room temperature for 10 min.

3) Add 1.7 ml of filter-sterilized 2M glycine to terminate cross-linking; incubate at room temperature for 5 min. Invert several times.

4) Filter lysate through 1 layer of miracloth into a clean centrifuge tube on ice.

5) Pellet the nuclei at 3000g for 20 min at 4˚C to obtain a white or nearly white pellet with an overlay of dissolved chlorophyll.

6) Discard supernatant and re-suspend the pellet in 300µl cold NIB (without Triton X-100, 2ME, Formaldehyde and protease inhibitor tablet). Avoid pipetting up and down as this may disrupt the nuclear membrane. Instead, re-suspend pellet by shaking gently.

7) Transfer the nuclear suspension into a 15% Percoll Solution.* Centrifuge at 3000g for 5min at 4˚C. (15% Percoll will separate the nuclei from the carried over chloroplasts). Discard supernatant; the chromatin is the white pellet at the bottom.

8) Add 600µl cold Nuclear Lysis Buffer and re-suspend the pellet with pipet and vortex vigorously.

9) Set aside 5 µl to compare with sonicated samples by gel electrophoresis (step 10).

10) Sonicate the lysate (*e.g. Branson 150, 7 times for 10 s at power 2; Model 100 Sonic Dismembrator; Fisher Scientific, 5 times for 15 s at power 6; etc.*) Cool samples on ice between pulses and avoid foaming.

11) Remove debris by spinning at top speed for 10 min at 4°C in microcentrifuge.

12) Transfer supernatant to new tube. If supernatant is not clear spin again and remove supernatant to new tube. Remove 5 µl to compare with the aliquot from step 7. Resolve on a 1% agarose gel. Reverse cross-linking the sonicated sample will show a smear from 200-1000 bp, but concentrated around 500 bp.

PAUSE POINT. Sonicated chromatin can be frozen at -80°C for 3 months.

***Solutions**

Nuclei Isolation Buffer (Keep Cold until Use) **<u>25ml</u>**

10mM HEPES pH 7.6	250µl 1M Hepes pH 7.6
1M sucrose	21.37ml 1.17M Sucrose
5mM KCl	125µl 1M KCl
5mM MgCl2	125µl 1M MgCl2
5mM EDTA	250µl 0.5M EDTA pH 8.0
PCR H2O	1.405 ml PCR H2O

To the 25ml NIB add the below right before starting the assay:

1% Formaldehyde; 700µl of 37% formaldehyde
14mM β-ME (commercial β-ME is about 14M; 25µl in 25ml)
0.6% Triton X-100 (750µl filter-sterilized 20% Triton X-100 in 25ml)
1/2 of a large protease inhibitor tablet (for 25ml of buffer)

Percoll Solution (Keep Cold until Use) **<u>25ml</u>**

15% Percoll	3.75ml Percoll
10mM HEPES pH 7.6	250ul 1M Hepes[#] pH 7.6
1M sucrose	12.5ml 2M Sucrose
5mM KCl	125ul 1M KCl
5mM MgCl2	125ul 1M MgCl2
5mM EDTA	250ul 0.5M EDTA pH 8.0
PCR H2O	8 ml PCR H2O

Nuclei Lysis Buffer (Keep at Room Temperature) **<u>5ml</u>**

50mM Tris-HCl pH 7.5	500ul 0.5M Tris-HCl pH 7.5
1% SDS	250ul 20% SDS
10mM EDTA	100ul 0.5M EDTA pH 8.0
PCR H2O	4.150ml PCR H2O

1/2 of a mini protease inhibitor tablet (for 5ml of buffer)

Chromatin Immunoprecipitation Protocol PART II

1) Transfer 10µl sonicated chromatin to a clean tube to serve as the 'input DNA control.' Store -80 °C until step 16. Transfer 100µl sonicated chromatin to a clean tube to serve as 'sonication efficiency control.' Store at -80 °C until step 16.

2) Add 1000µl ChIP Dilution Buffer to each of two 150µl-aliquots of sonicated chromatin (one of these will receive pre-immune serum and will serve as a negative control), in a 2ml microfuge tube. It is essential to dilute the nuclear lysate at least 10 times to inactivate the SDS. If the SDS concentration is greater than 0.1%, it is likely that the antibody will become denatured.

3) To reduce background caused by non-specific absorption of irrelevant proteins to Protein A-agarose, add 50ul Protein A-Agarose beads to both tubes. Incubate 3hr to overnight at 4°C with gentle rotation.

4) Pellet agarose beads by centrifugation at 12,000g for 30 seconds at 4°C. Transfer supernatant (S/N) to a fresh 2ml microfuge tube. Discard beads (pellet).

5) Add x µl (5µg) antibody to only one of the tubes; and pre-immune serum to the negative control. Incubate 2hr to overnight at 4°C with gentle rotation.

6) Add 50µl Protein A-Agarose beads to both tubes. Incubate 3 hr to overnight at 4°C with gentle rotation.

7) Collect Protein A-Agarose-antibody-antigen complexes by centrifugation for 30 sec at 3,800g at 4°C. Carefully remove supernatant, re-suspend pellet in 1ml Wash Buffer 1,* spin beads 30 sec 3,800g at 4°C, remove S/N, re-suspend pellet in 1ml Wash Buffer 1 and agitate 5 minutes at 4°C.

8) Spin 30 sec at 3,800g at 4°C. Carefully remove supernatant, re-suspend pellet in 1ml Wash Buffer 2,* spin beads 30 sec at 3,800g at 4°C, remove S/N and re-suspend pellet in 1ml Wash Buffer 2 and agitate 5 minutes at 4°C.

9) Spin 30 seconds at 3,800g at 4°C. Carefully remove supernatant, re-suspend pellet in 1ml Wash Buffer 3*, spin beads 30 seconds 3,800g at 4°C, remove S/N re-suspend pellet in 1ml Wash Buffer 3 and agitate 5 minutes at 4°C.

10) Spin 30 seconds at 3,800g at 4°C. Carefully remove supernatant, re-suspend pellet in 1ml Wash Buffer 4,* spin beads 30 seconds at 3,800g at 4°C, remove S/N, re-suspend pellet in 1ml Wash Buffer 4 and agitate 5 minutes at 4°C.

11) Spin 30 seconds at 3,800g at 4°C. Carefully remove the last traces of the TE from the agarose pellet and from the walls and lid of the microcentrifuge tube.

12) To elute the immuno-complexes from the beads, add 250µl Elution Buffer,* and vortex briefly and incubate at 65^{0}C for 15 minutes with gentle agitation by inverting tube every 3 min.

13) Spin at 3,800g for 2 min at room temperature and transfer the S/N to a new 1.5-ml tube.

14) Repeat the elution steps (steps 12 and 13) by adding another 250µl Elution Buffer to the beads.

15) Combine the eluates from steps 12 and 13 (500µl total).

16) Add 490µl Elution Buffer* to the 10µl sonicated chromatin reserved in step 1 to make the input DNA control. Add 900µl Elution Buffer* to the 100µl sonicated chromatin reserved in step 1 to test sonication efficiency.

17) Add 20µl 5M NaCl to the samples and incubate at 65^{0}C for 4 hr to overnight to reverse cross-linking.

18) Add Proteinase K solution to each sample (10µl 0.5M EDTA pH8.0, 20µl 1M Tris-HCl pH6.5 and 20µg Proteinase K) and incubate at 50°C for 2hr.

19) Bring samples and phenol/chloroform to room temperature.

20) Add 520µl phenol:chloroform to each sample; vortex; incubate on shaker at room temperature for 5 min and spin 15 min high speed at 4°C.

21) Transfer 400µl of the aqueous layer (top layer) to a new tube and add 50µl 3M NaOAc pH 5.2, 2µl glycogen, and 1ml cold 100% EtOH; incubate at -20°C at least 30 min to overnight.

22) Spin 20 min at top speed at 4°C; remove S/N; add 75% EtOH; spin 10 min.

23) Remove S/N and dry pellet at 30°C

24) Dissolve the DNA in 30µl 10 mM Tris-HCl and 60µg/ml Rnase A . Dilute RNase A 1/10 dilution (1µl RNAse A at 29mg/ml + 9ul PCR H_2O). Add 0.3µl diluted RNase A to each sample. Incubate at 37⁰C 30 min and store at -80°C.

Determination of OsH2A.Z Antibody Specificity

An N-terminal peptide specific to OsH2A.Z was used to synthesize a polyclonal anti-OsH2A.Z antibody (Figure 1a). To test the antibody on recombinantly expressed OsH2AZ, the vector pET15b was used for expression of *HTA713*. Diagnostic restriction digestion analysis and sequencing confirmed that the proper plasmid was made. The plasmid was introduced into E. coli and expression was induced with IPTG (100mM). Crude protein extracts were separated via SDS-PAGE gel electrophoresis. After transferring to a PVDF membrane, the rice H2A.Z antibody serum was used for immunoblotting. The antibody reacted with the HTA713 protein (Figure 1b, lane 1). Next, rice protein extracts from young leaves were resolved with SDS-PAGE and transferred to a PVDF membrane. Immunoblotting detected a band with the appropriate size for HTA713 using serum but not pre-immune serum (Figure 1b, lanes 3-6).

Immunoblotting analysis

100 mg of frozen seedlings was ground to a fine powder in liquid nitrogen, and extracted proteins mixed with loading buffer were boiled for 5 min (in the case of recombinantly expressed *HTA713*, 50 µl sample were mixed with 50 µl loading buffer) and spun for 10 min to remove cellular debris. Mini-Protein Precast gels (Bio-Rad) were used for SDS-PAGE to separate proteins, and then blotted onto polyvinylidene difluoride (PVDF) membranes (Bio-Rad). The membranes were blocked in 5% blocking buffer (milk +Tris Buffered Saline with Tween 20 (TBST) at room temperature for 2h following 3 5min washes with TBST. The membranes were incubated overnight at 4°C with OsH2A.Z antibody or pre-immune serum in Tris Buffered Saline with BSA (TBSB) (primary antibody). After washing 5 times in TBST for 5 min, the membranes were incubated for 2 h at room temperature with 1/5000 diluted HSP-conjugate goat anti-rabbit (secondary antibody). The membranes were washed 5 times for 5 min in TBST and incubated for 1 min in the mixture of SuperSignal West Pico Chemiluminescence Substrate (34077; ThermoFisher Scientific). The signals were detected by molecular imager ChemiDoc XRS+ (Bio-Rad).

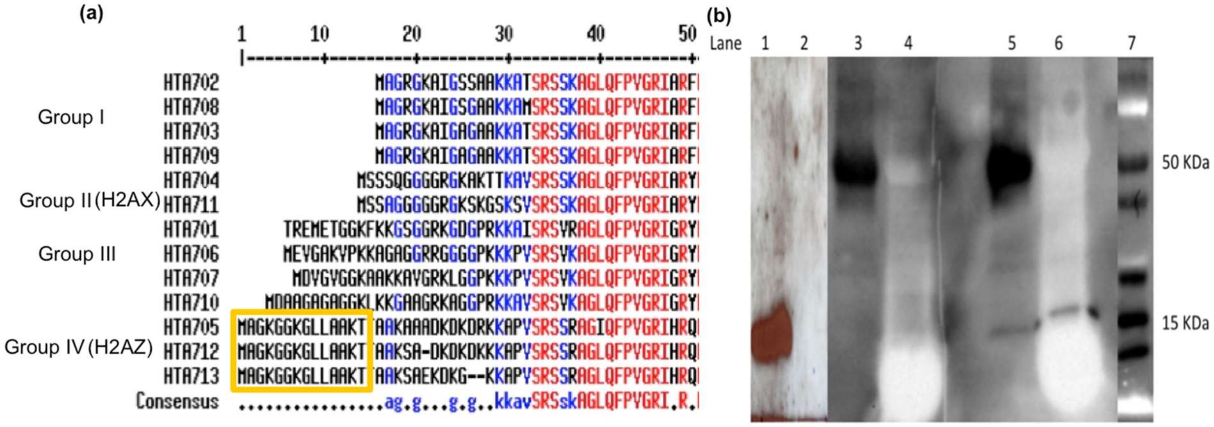

Figure 1. Determination of OsH2A.Z Antibody Specificity. (a) N-terminal 50 amino acids alignment of the H2A family using the online Multalin tool. Red bases are identical in all aligned sequences, blue shows the most frequent amino acid in aligned sequences and black shows a lack of high similarity among all sequences. The sequence in the yellow box was selected for peptide synthesis. (b) Immunoblotting using the OsH2A.Z antibody to test its specificity. Lanes 1 and 2: Protein extracts from *E. coli* expressing recombinant HTA713 were made from cultures 4 hours after IPTG induction (Lane 1), or without induction (Lane 2). Lanes 3 and 5: Immuno-precipitated proteins, pre-immune serum used as a negative control in an immunoblot assay (Lane 3) and OsH2A.Z crude serum (Lane 5). Lanes 4 and 6: Protein extracts from rice shoots. Pre-immune serum used as a negative control in immunoblot assay (Lane 4) and OsH2A.Z crude serum (Lane 6). The expected size for OsH2A.Z is 15 KDa and expected size for IgG present in the serum is 50 KDa. Lane 7, size standard.

References Cited

Smith, A. P., Jain, A., Deal, R. B., Nagarajan, V. K., Poling, M. D., Raghothama, K. G., & Meagher, R. B. (2010). Histone H2A.Z regulates the expression of several classes of phosphate starvation response genes but not as a transcriptional activator. Plant Physiology, 152(1), 217-225.

Widiez, T., Symeonidi, A., Luo, C., Lam, E., Lawton, M., & Rensing, S. A. (2014). The chromatin landscape of the moss Physcomitrella patens and its dynamics during development and drought stress. The Plant Journal, 79(1), 67-81.
